# Single-cell and bulk RNA sequencing reveal cancer-associated fibroblast heterogeneity and a prognostic signature in prostate cancer

**DOI:** 10.1097/MD.0000000000034611

**Published:** 2023-08-11

**Authors:** Wen Liu, Miaomiao Wang, Miao Wang, Ming Liu

**Affiliations:** a Department of Urology, Beijing Hospital, National Center of Gerontology, Institute of Geriatric Medicine, Chinese Academy of Medical Sciences, Beijing, China; b Graduate School of Peking Union Medical College, Chinese Academy of Medical Sciences, Beijing, China.

**Keywords:** cancer-associated fibroblast, prognostic signature, prostate cancer, single-cell

## Abstract

Cancer-associated fibroblasts (CAFs), the central players in the tumor microenvironment (TME), can promote tumor progression and metastasis via various functions. However, the properties of CAFs in prostate cancer (PCa) have not been fully assessed. Therefore, we aimed to examine the CAF characteristics in PCa and construct a CAF-derived signature to predict PCa prognosis. CAFs were identified using single-cell RNA sequencing (scRNA-seq) data from 3 studies. We performed the FindAllMarkers function to extract CAF marker genes and constructed a signature to predict the biochemical relapse-free survival (bRFS) of PCa in the Cancer Genome Atlas (TCGA) cohort. Subsequently, different algorithms were applied to reveal the differences of the TME, immune infiltration, treatment responses in the high- and low-risk groups. Additionally, the CAF heterogeneity was assessed in PCa, which were confirmed by the functional enrichment analysis, gene set enrichment analysis (GSEA), and AUCell method. The scRNA-seq analysis identified a CAF cluster with 783 cells and determined 183 CAF marker genes. Cell-cell communication revealed extensive interactions between fibroblasts and immune cells. A CAF-related prognostic model, containing 7 genes (ASPN, AEBP1, ALDH1A1, BGN, COL1A1, PAGE4 and RASD1), was developed to predict bRFS and validated by 4 independent bulk RNA-seq cohorts. Moreover, the high-risk group of the signature score connected with an immunosuppressive TME, such as a higher level of M2 macrophages and lower levels of plasma cells and CD8^+^ T cells, and a reduced reaction rate for immunotherapy compared with low-risk group. After re-clustering CAFs via unsupervised clustering, we revealed 3 biologically distinct CAF subsets, namely myofibroblast-like CAFs (myCAFs), immune and inflammatory CAFs (iCAFs) and antigen-presenting CAFs (apCAFs). In conclusion, the CAF-derived signature, the first of its kind, can effectively predict PCa prognosis and serve as an indicator for immunotherapy. Furthermore, our study identified 3 CAF subpopulations with distinct functions in PCa.

## 1. Introduction

Prostate cancer (PCa) is the second most common malignancy among males worldwide.^[1]^ Although curative treatment generally leads to a good prognosis for nonmetastatic PCa, biochemical recurrences (BCR) occur in approximately 30% of the patients.^[2]^ PCa is a heterogeneous disease with varied prognoses, and a combination of clinical and pathological information cannot fully elucidate the tumor behavior.^[3]^ Thus, it is necessary to identify independent prognostic biomarkers for BCR risk stratification. Moreover, aggressive and individualized treatment for high-risk patients might improve the prognosis.

It has been gradually recognized that cancer progression and metastasis depend not only on tumor cells but also on the interconnection and dependence of the tumor stroma.^[4]^ Cancer-associated fibroblasts (CAFs), the central players in the tumor microenvironment (TME), stimulate extracellular matrix (ECM) remodeling, cancer cell growth, and angiogenesis, leading to a worse prognosis.^[5–7]^ CAFs can promote cancer invasion through direct contact with other cell types, thereby regulating cancer cell migration via activation of the Eph-Ephrin signaling.^[8]^ CAFs also indirectly modulate cancer progression by secreting contractile proteins (ACTA2, TAGLN) and ECM components (DCN, LUN, collagen).^[9,10]^ Moreover, CAFs contribute to immunosuppression and immune evasion in the TME by regulating the immune components and cells.^[5]^ Considering the prognostic significance of CAFs, identifying prognostic-related CAF genes may help distinguish patients at high risk for progression.

Based on single-cell RNA sequencing (scRNA-seq) technologies, the heterogeneity of CAFs has been well recognized in several cancers, such as breast,^[11]^ pancreatic,^[12]^ lung,^[13]^ and ovarian^[14]^ cancers. Three broad CAF subpopulations have been summarized in these cancers, namely myofibroblastic CAFs, pro-inflammatory and immune-regulatory CAFs, and antigen-presenting CAFs (apCAFs).^[9]^ Investigating the distinctive properties of these CAF subsets may uncover the therapeutic resistance mechanisms and may lead to the development of CAF-targeting strategies.^[15]^ In PCa, Chen et al found that most CAFs presented myofibroblastic-like signatures,^[16]^ whereas Vickman et al identified inflammatory prostate CAF subsets.^[17]^ Therefore, we postulated that a similar CAF classification may exist in PCa as in breast and pancreatic cancers.

Compared with bulk RNA sequencing (bulk RNA-seq), scRNA-seq can help distinguish CAFs and discover their biological diversity. Several recent studies have revealed the transcriptome landscape in PCa based on scRNA-seq methods, but the properties of CAFs have not been fully assessed.^[16,18,19]^ Therefore, in this study, we integrated the scRNA-seq data from the above-cited 3 studies to comprehensively elucidate the functions and heterogeneity of CAFs. We also established a CAF-derived signature to predict the BCR of patients with PCa using bulk RNA-seq data.

## 2. Methods

### 2.1. Data source

The scRNA-seq data related to PCa were downloaded from GSE141445,^[16]^ GSE157703,^[19]^ and GSE185344^[18]^ via the Gene Expression Omnibus (GEO) database (https://www.ncbi.nlm.nih.gov/geo/). After excluding 4 samples from metastatic patients and 2 samples that lacked fibroblasts, a total of 7 samples were included from GSE141445. In addition, GSE157703 and GSE185344 contained 2 and 7 primary tumors undergoing prostatectomy, respectively. Finally, scRNA-seq analysis was based on these 16 samples. The bulk transcriptome data and clinical information of 491 patients with PCa were obtained from The Cancer Genome Atlas (TCGA) database (https://portal.gdc.cancer.gov/). After removing 83 patients owing to metastases or a lack of clinical or survival data, 408 patients with PCa were selected for further signature construction. To validate the signature, 4 datasets were downloaded from Memorial Sloan-Kettering Cancer Center (MSKCC) (n = 131) and GEO databases, including GSE54460 (n = 97), GSE116918 (n = 248), GSE46602 (n = 36). All datasets were publicly downloadable, and patient consent and ethical approval were obtained in the original studies.

### 2.2. Single-cell RNA-seq data analysis

We used the Seurat v4.1.3 package to process scRNA-seq data.^[20,21]^ Several quality measures were applied: cells with fewer than 200 features (low quality) or >8000 features (doublets/multiplets) were removed; cells with more than 20% mitochondrial genes or 5% hemoglobin genes were removed (low quality) (Supplementary Fig. 1A–B, http://links.lww.com/MD/J443). The gene expression of the remaining cells was normalized using the NormalizeData function. Cells from different samples were integrated using the canonical correlation analysis method in the Seurat package to remove batch effects and to ensure that the cells were comparable.^[21]^ Based on the batch-corrected data, highly variable genes (top 2000) were scaled and utilized to perform principal component analysis (PCA). Subsequently, the shared nearest neighbor method and Louvain algorithm were performed for non-supervised clustering using the top 50 principal components (resolution = 1.0), and the Uniform Manifold Approximation and Projection was conducted to visualize the 32 clusters (Supplementary Fig. 1C–D, http://links.lww.com/MD/J443). We manually annotated the clusters based on canonical marker genes. Additionally, the FindAllMarkers and FindMarkers functions were used to identify the differentially expressed (marker) genes (DEGs) for clusters with default parameters. The significant DEGs of fibroblasts were filtered as those with log2 fold change ≥ 1 and adjusted *P* values < .05.

### 2.3. Construction and validation of the CAF-related prognostic signature

CAF-related marker genes were extracted to develop a risk prognostic signature. Firstly, univariate Cox regression analysis was performed using the TCGA cohort to identify the CAF-related genes significantly associated with the BCR of PCa (*P <* .05). Next, the LASSO regression analysis was employed to minimize the overfitting risk of the prognostic model.^[22]^ Based on the result of multivariable COX regression analysis, the signature was calculated as follows: $Risk\;score=\overset{n}{\underset{i=1}{\sum }}\beta_{i}\times Exp_{i}$, where $\beta_{i}$ is the risk coefficient, and $Exp_{i}$ represents the mRNA expression of each selected gene. The median risk score was considered the cutoff to classify patients into high- and low-risk groups. Moreover, the Kaplan–Meier (KM) and Log-rank methods were used to assess the difference in the biochemical relapse-free survival (bRFS) between the 2 risk groups. The receiver operating characteristic (ROC) curve was generated to evaluate the predictive accuracy of the CAF-related signature. We also extracted the coefficients for the selected genes to validate the CAF-derived signature in the MSKCC, GSE54460, GSE116918, and GSE46602. The R packages survival, survminer, rms, and timeROC were used for these analyses.

### 2.4. Nomogram construction and validation

Multivariate Cox regression analysis was performed to investigate whether the risk score was an independent predictor in PCa. Then, a nomogram was developed based on the results of Cox regression analyses to predict 1-, 3-, and 5-year bRFS probabilities. To evaluate the nomogram predictive performance and consistency, we calculate the concordance index (C-index) and visualize the calibration curve, respectively.

### 2.5. Functional and gene set enrichment analysis (GSEA)

To explore the potential function of the identified cluster, the “Clusterprofiler” package was employed to perform gene ontology (GO) and the Kyoto Encyclopedia of Genes and Genomes (KEGG) analysis using the marker genes.^[23]^ Additionally, GSEA was performed using the Hallmark and KEGG gene sets from The Molecular Signatures Database to identify the significant gene sets among the CAF subsets.^[23]^ An adjusted *P* < .05 was considered statistically enriched.

The “area under the curve” (AUC) method can calculate the AUCell scores of single cells based on the input gene signature. A higher AUCell score in a single cell represents a more enriched activity of the gene set of interest.^[24,25]^ We searched for related gene sets of the CAFs and macrophage polarization from the published studies to calculate the AUCell scores.^[12,24,26]^

### 2.6. TME estimation

The fibroblast infiltration levels were calculated using the xCell, Microenvironment Cell Populations counter (MCP-counter), and Estimate the Proportion of Immune and Cancer cells (EPIC) algorithms.^[27–29]^ Additionally, the estimation of stromal and immune cells in malignant tumor tissues using expression data (ESTIMATE) and xCell algorithms were applied to estimate each patient stromal and immune scores.^[28,30]^ A signature score was also alculated based on all fibroblast marker genes using gene set variation analysis.^[31]^ We used the Cell-type Identification By Estimating Relative Subsets Of RNA Transcripts (CIBERSORT) algorithm to assess the correlation of 22 immune cells with risk scores, selected genes, and the signature of all CAF marker genes.^[32]^

### 2.7. Prediction of the response to treatment

Using Tumor Immune Dysfunction and Exclusion (TIDE) algorithm, we calculated the TIDE score to assess the tumor immune evasion potential.^[33]^ A high TIDE score indicates a higher chance of immune evasion and poorer response to immunotherapy. We also compared the expression levels of immune checkpoint molecules, such as programmed cell death protein 1 (PD-1), programmed death receptor ligand-1 (PD-L1), and cytotoxic T lymphocyte associate protein-4 (CTLA4), in 2 groups. The half-maximal inhibitory concentration (IC50) was calculated to measure the sensitivity to chemotherapy in patients with PCa (pRRophetic R package).^[34]^

### 2.8. Trajectory analysis and cell communication analysis

We performed a pseudotime trajectory analysis to investigate the evolutionary characteristics of the CAF subsets using the Monocle 2 algorithm.^[35]^ Cell-cell communication analyses were conducted using the “Cellchat” R package to explore underlying interactions between CAFs and other cells, especially immune cells.^[36]^ According to the reference communication network, the probability of cell interaction in the pathway was inferred from the ligand and receptor gene expression levels.

### 2.9. Immunohistochemical (IHC) and immunofluorescent (IF) staining

We assessed the expression levels of CAF signature genes in prostatectomy samples using IHC staining obtained from the Human Protein Atlas (HPA) database (https://www.proteinatlas.org).^[37]^ In addition, we performed IHC and IF staining on prostatectomy samples obtained from the Beijing Hospital. For IHC staining, primary antibodies raised in rabbit against AEBP1 (Sigma Cat# HPA064970, RRID:AB_2685394, diluted 1:20) and PAGE4 (Sigma Cat# HPA023880, RRID:AB_1854930, diluted 1:500) were used, followed by detection using the Rabbit Kit-9902 (MaiXin Reagent), a HRP-linked secondary antibody raised in goat against rabbit IgG. For IF staining, we used primary antibodies raised in rabbit against COL1A1 (Abcam Cat# ab138492, RRID:AB_2861258, diluted 1:2000) and BGN (Abcam Cat# ab209234, RRID:AB_2861218, diluted 1:1000) to detect the expression levels in tissue. The sections were then incubated with a goat anti-rabbit IgG secondary antibody (ZF-0316, Zhongshan Golden Bridge), followed by TRITC-conjugated red fluorescent dye (Zhongshan Golden Bridge) for visualization. The samples of PCa were collected in accordance with the regulations of the Ethics Committee of Beijing Hospital (2023BJYYEC-068-01), and informed consent was obtained from all patients.

### 2.10. Statistical analysis

Statistical analyses were performed using R version 4.1.2. Continuous variables between the 2 groups were compared using the Wilcoxon-rank sum test, while the Spearman correlation analysis was used to evaluate the correlation between continuous variables. A *P* value <.05 was considered statistically significant in all analyses.

## 3. Results

### 3.1. Composition of PCa at cellular resolution

**Figure 1.**
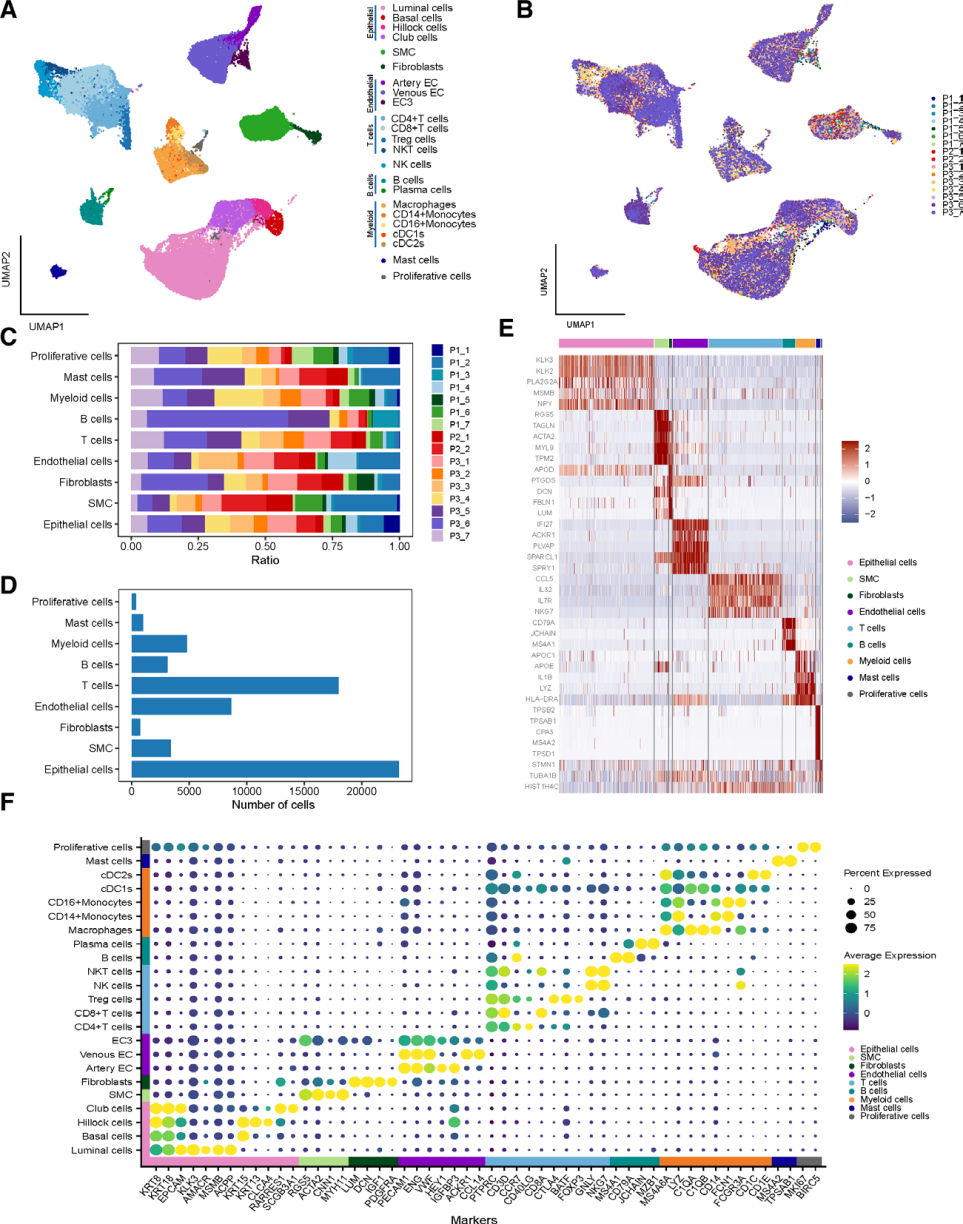
Single-cell RNA sequencing analysis of 63,245 cells from patients with PCa. (A) Twenty-three cell types are annotated based on UMAP algorithm and marker genes. (B) UMAP visualization is delineated by the patient origin. (C) Proportion of cell types across 16 patients. (D) Number of cells in different cell types. (E) Heatmap presents the top 5 marker genes in different cell types. (F) Bubble plot of canonical marker genes used for distinguishing cell types. PCa = prostate cancer, UMAP = Uniform Manifold Approximation and Projection.

Nine independent cell clusters were distinguished using Seurat and there was no apparent heterogeneity of cell types across patients (Fig. 1A–C). Post quality control, a total of 63,245 cells were analyzed in the scRNA-seq data (Fig. 1d). Cell clusters were annotated using canonical marker genes for epithelial cells (EPCAM, KRT18), smooth muscle cells (RGS5), fibroblasts (LUM, PDGFRA), endothelial cells (PECAM1), T cells (CD3D), B cells (MS4A1), myeloid cells (MS4A6A), mast cells (TPSAB1), and proliferative cells (MKI67) (Fig. 1E and Supplementary Fig. 1E, http://links.lww.com/MD/J443). A more detailed classification and related canonical genes are presented in Figure 1f.

### 3.2. Identification of marker genes and biological pathways in CAFs

**Figure 2.**
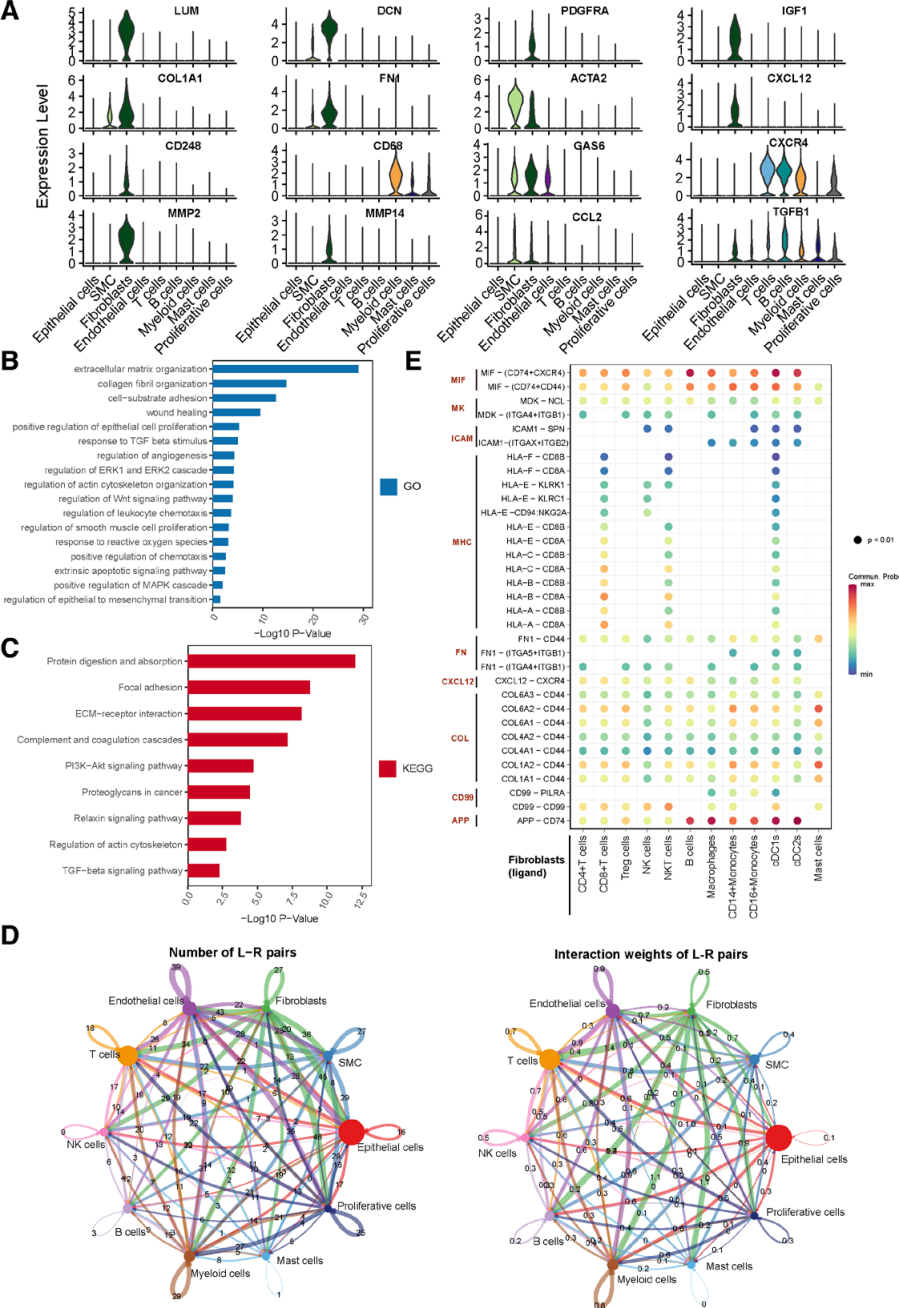
The functional enrichment analysis of CAFs and cell communication analysis. (A) Violin plots of gene expression in different cell types. (B–C) GO (B) and KEGG (C) analyses of CAF marker genes. (D) Overall intercellular communication profiles between each cell type in PCa. The thickness of the line indicates the number or interaction weights of ligand-receptor pairs. (E) Bubble plot shows pathways and ligand-receptor pairs between fibroblasts and immune cells. CAFs = cancer-associated fibroblasts, GO = gene ontology, KEGG = Kyoto Encyclopedia of Genes and Genomes, PCa = prostate cancer.

In total, 738 CAFs were found to express the common fibroblast-specific markers, including LUM, DCN, PDGFRA, IGF1, and COL1A1, confirming the cluster annotation (Fig. 2A).^[18,38,39]^ Furthermore, 183 marker genes were identified in the CAFs compared with those in the other clusters (Supplementary Table 1, http://links.lww.com/MD/J444). GO and KEGG analyses were conducted to explore the biological functions of CAFs using these marker genes (Fig. 2B–C). The results were enriched in terms including collagen fibril organization, ECM organization, epithelial cell proliferation, angiogenesis, epithelial-to-mesenchymal transition (EMT), wound healing, leukocyte chemotaxis, and complement and coagulation cascades. This suggested that CAFs located in the TME regulated the immune system and cancer cell growth, which could promote tumor progression and metastasis.^[15,40]^ The cell-cell communication revealed extensive interactions between fibroblasts and other cell types (Fig. 2D and Supplementary Fig. 2A–C, http://links.lww.com/MD/J445). Moreover, the fibroblasts could communicate with immune cells through the macrophage migration inhibitory factor (MIF), Midkine (MK), major histocompatibility complex (MHC), Fibronectin (FN), C-X-C chemokine ligand 12 (CXCL12), Collagen (COL), Amyloid Beta Precursor Protein (APP) signaling pathways (Fig. 2E).

### 3.3. Construction of CAF-derived prognostic signature

**Figure 3.**
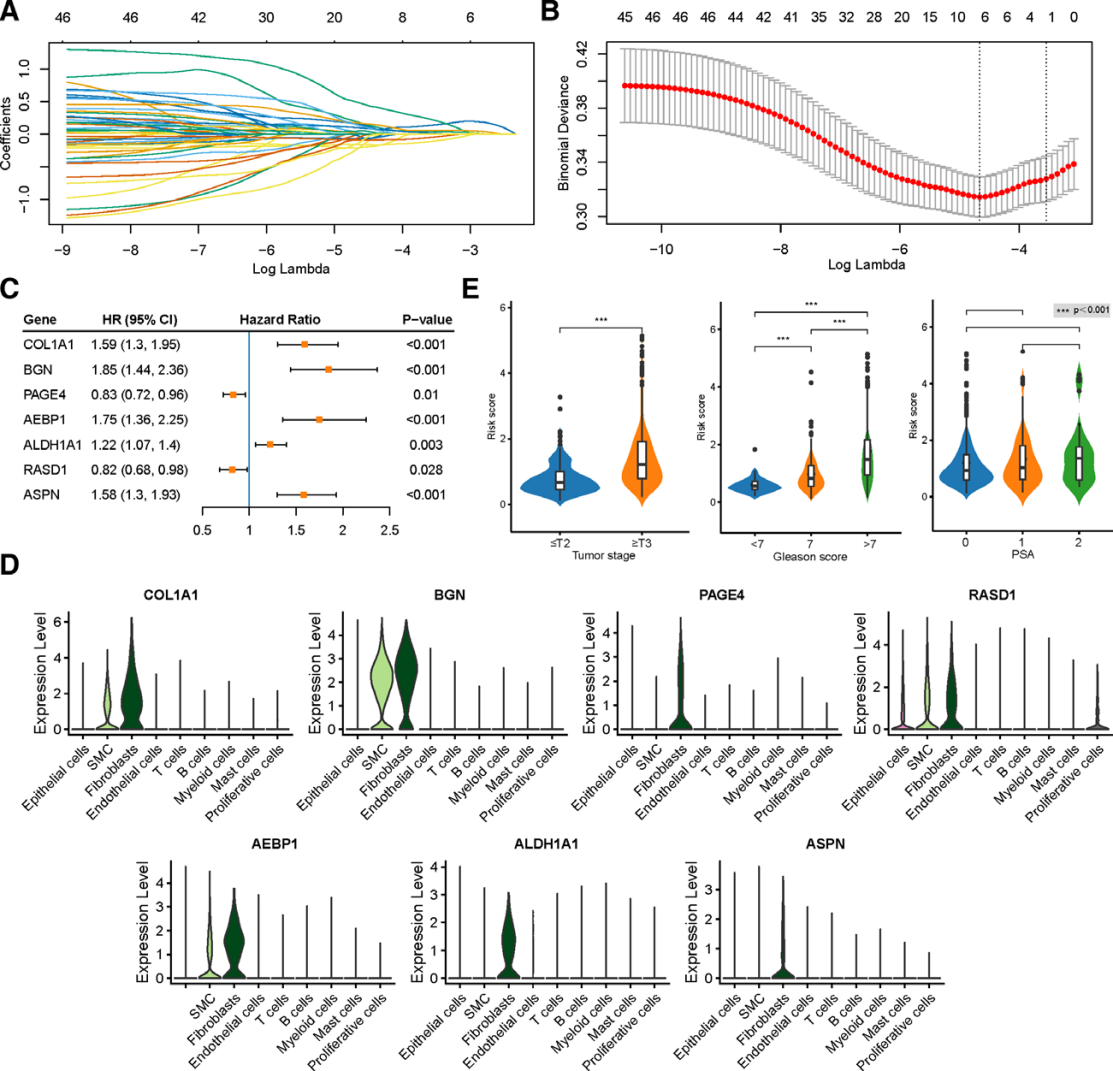
The construction of CAF-related prognostic signature in the TCGA cohort. (A–B) A prognostic signature containing 7 genes is constructed by LASSO Cox regression analysis. (C) Univariate cox regression analysis of 7 candidate genes, including 5 risk genes and 2 protective genes. (D) Differences of risk scores in tumor stage, grade score, and prostatic specific antigen (PSA). (E) Violin plots show the expression of 7 candidate genes in different cell types. CAFs = cancer-associated fibroblasts, TCGA = The Cancer Genome Atlas.

**Figure 4.**
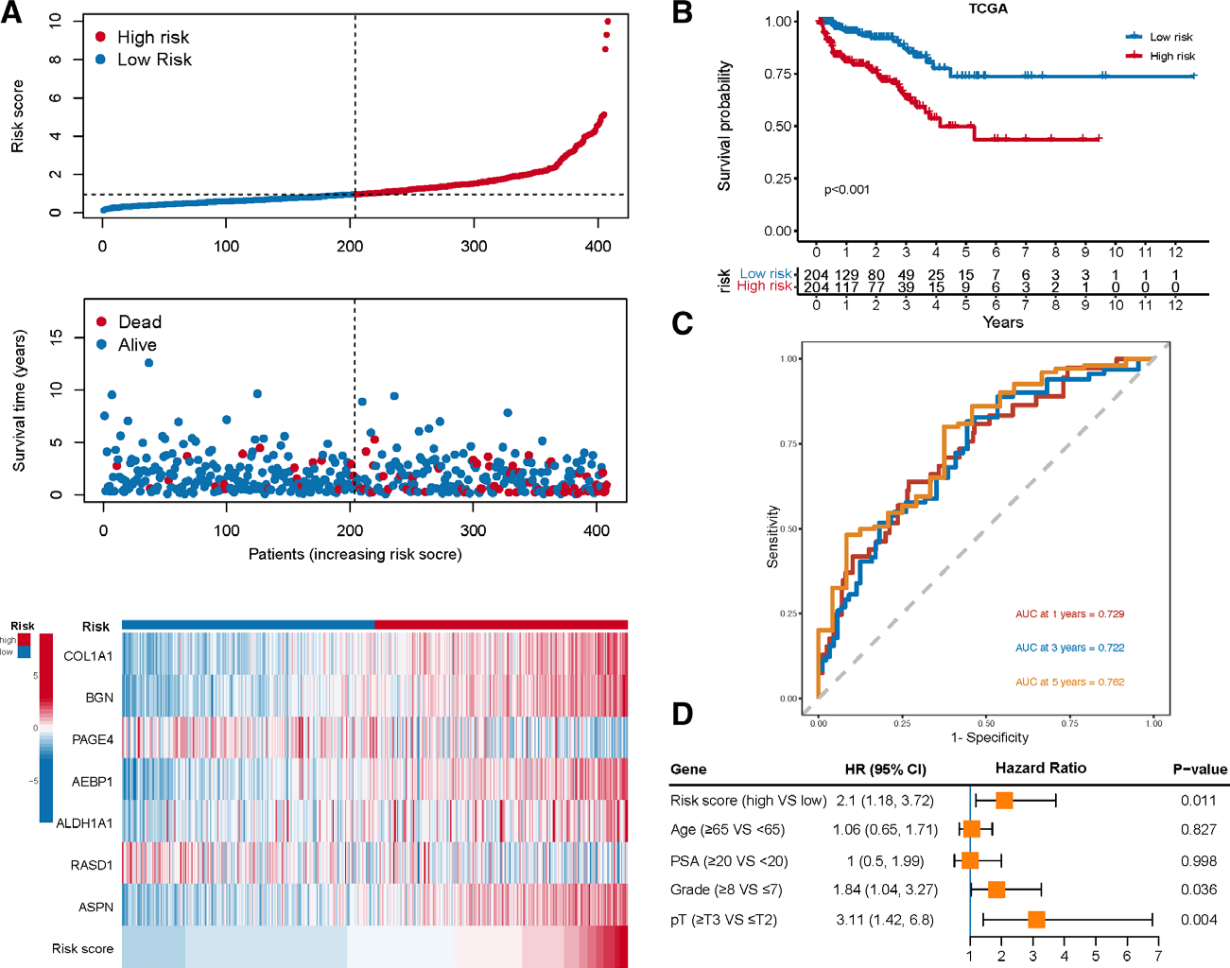
The evaluation of prognostic signature in the TCGA cohort. (A) The distribution of risk score, survival status, and gene expression across patients with PCa. (B) KM survival curve of biochemical relapse-free survival (bRFS) between high-risk and low-risk groups. (C) Receiver operating characteristic curve (ROC) analysis predicts the accuracy of 3-, 4-, and 5-yr bRFS in the CAF-derived prognostic model. (D) Multivariate Cox regression analysis shows that the risk score independently increased the risk of biochemical recurrence in patients with PCa. CAFs = cancer-associated fibroblasts, KM = Kaplan–Meier, PCa = prostate cancer, TCGA = The Cancer Genome Atlas.

As CAFs can promote tumor progression,^[9]^ we used the CAFs marker genes to construct a prognostic signature using the TCGA cohort. Through univariate Cox regression analyses, we identified 46 CAF-related marker genes that were significantly associated with the bRFS (*P* < .05, Supplementary Table 2, http://links.lww.com/MD/J446). The LASSO and multivariable cox regression analyses were used to determine the most valuable predictive genes, yielding a prognostic signature of 7 genes, comprising 5 risk genes and 2 protective genes (Fig. 3A–C and Supplementary Fig. 3A, http://links.lww.com/MD/J447). The risk score of gene signature was calculated via the following formula: Risk score=0.062×COL1A1+0.033×BGN−0.216×PAGE4+0.485×AEBP1+0.067×ALDH1A1−0.147×RASD1+0.066×ASPN. As shown in the violin plot, all 7 marker genes were highly expressed in the fibroblasts (Fig. 3D). The results of IHC and IF staining in PCa samples displayed that the candidate genes were highly expressed in the stromal cells of PCa tissue (Supplementary Fig. 4A–K, http://links.lww.com/MD/J448). Subsequently, we divided the samples into high- and low-risk groups based on the median score and found that increased risk scores were associated with a higher tumor stage and Gleason score (GS) (Fig. 3E). The distribution of risk scores and survival status in all samples are presented in Figure 4A. With regard to the prognosis of PCa, the KM curve showed that a high-risk score significantly increased the risk of bRFS (*P* < .001, Fig. 4B). The ROC curves revealed that the AUC of 1-, 3-, and 5-year survival was 0.729, 0.722, and 0.762, respectively, proving the predictive accuracy of the CAF-derived signature (Fig. 4C). Moreover, the multivariate Cox regression model revealed that the risk group was an independent prognostic risk factor in PCa (HR 2.1, 95%CI 1.18–3.72; Fig. 4D).

### 3.4. Validation of the prognostic signature and nomogram construction

**Figure 5.**
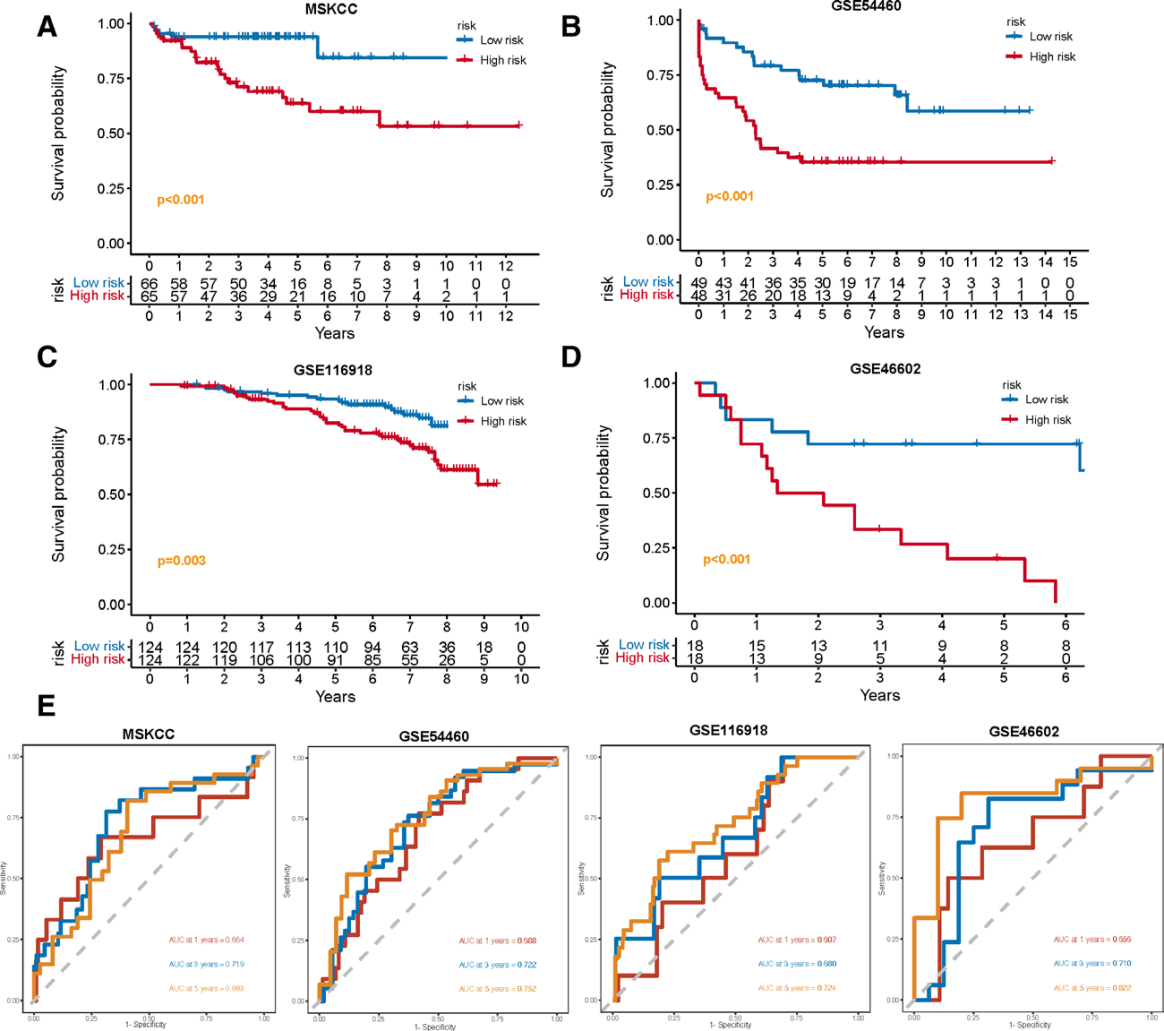
The validation of CAF-related prognostic signature in 4 independent cohorts. (A–D) KM survival curves of biochemical relapse-free survival (bRFS) between high-risk and low-risk groups in the validation cohorts, including MSKCC (A), GSE54460 (B), GSE116918 (C), and GSE46602 (F). (E) Receiver operating characteristic curve (ROC) analysis predicts the accuracy of 3-, 4-, and 5-yr bRFS in the CAF-derived prognostic model. CAFs = cancer-associated fibroblasts, KM = Kaplan–Meier.

Based on the constructed formula, we calculated the risk scores in 4 independent cohorts, including MSKCC, GSE54460, GSE116918, and GSE46602, and validated the predictive power of the CAF-related signature. The KM analysis in all 4 cohorts demonstrated that patients in the high-risk group had a worse prognosis (all *P* ≤ .003, Fig. 5A–D). Furthermore, the ROC curves verified that the prognostic signature had high predictive accuracy for 5-year BCR in MSKCC (AUC = 0.683), GSE54460 (AUC = 0.752), GSE116918 (AUC = 0.724), and GSE46602 (AUC = 0.822) (Fig. 5E).

A nomogram was established to better assess the prognosis of patients with PCa based on the risk scores (Supplementary Fig. 3B, http://links.lww.com/MD/J447). The C-index supported the predictive ability of the nomogram (C-index = 0.747) and the calibration plots showed good linearity between the actual and nomogram-predicted bRFS for the 1-, 3-, and 5-year survival rates (Supplementary Fig. 3C, http://links.lww.com/MD/J447). Together, this fundings indicate that the CAF-derived signature could serve as a reliable prognostic predictor for patients with PCa.

### 3.5. TME, immune infiltration, and M2 polarization of macrophages

**Figure 6.**
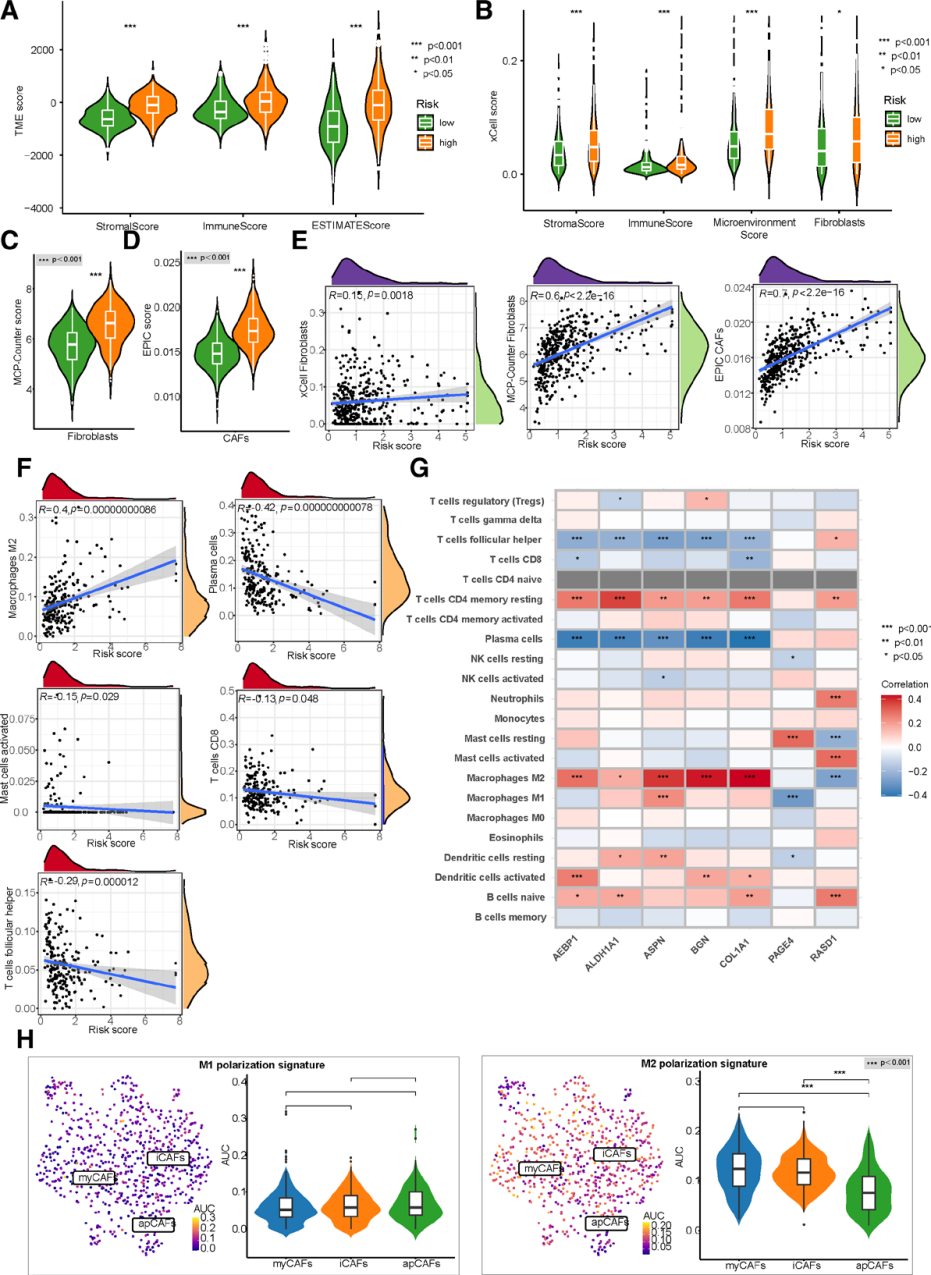
Correlation of the CAF-related prognostic signature with tumor microenvironment and immune infiltration. (A) Comparison of the stromal score, immune score, and ESTIMATE score between high-risk and low-risk groups using the ESTIMATE algorithm. (B) Comparison of the stromal score, immune score, microenvironment score, and fibroblast infiltration between high-risk and low-risk groups using the xCell algorithm. (C–D) Differences in fibroblast infiltration calculated by MCP-Counter (C) and EPIC (D) algorithms between high-risk and low-risk groups. (E) Three algorithms estimate the correlation between the risk score and fibroblast infiltration in PCa. (F) The risk score is positively associated with the proportion of M2 macrophages and negatively related to the infiltration of plasma cells, activated mast cells, CD8^+^ T cells, and follicular helper T cells. (G) Heatmap shows the correlation between the expression of 7 signature genes and 22 immune cells’ infiltration. (H) AUC values for fibroblasts are scored based on the published macrophage polarization signatures (M1 and M2 polarization). Meanwhile, violin plots show the differences in AUC scores among 3 CAF subsets (Azizi et al 2018). AUC = area under the curve, CAFs = cancer-associated fibroblasts, PCa = prostate cancer.

As a central constituent of TME in solid tumors, CAFs are responsible for cross-communications among immune cells, stromal cells, and tumor cells.^[15]^ In the current study, the high-risk group was associated with increased immune, stroma, and microenvironment scores based on analysis using the ESTIMATE and xCell algorithms (Fig. 6A–B). Moreover, using all 3 algorithms, namely xCell, MCP-Counter, and EPIC, we could verify that the risk score of the CAF-related signature was positively correlated with the fibroblast infiltration levels (Fig. 6B–E). With regard to immune cell infiltration, the CIBERSORT analysis demonstrated that patients with high-risk scores had a higher infiltration level of M2 macrophages, and lower levels of plasma cells, activated mast cells, CD8^+^ T cells, and follicular helper T cells (Fig. 6F). The 5 risk-related genes showed a consistent correlation with the immune infiltration compared to the 2 protective genes (Fig. 6G). Subsequently, we used gene set variation analysis to calculate a signature score based on all fibroblast marker genes. Notably, this CAF signature score was found to be associated with lower and higher levels of M1 and M2 macrophages, respectively, consequently shaping the immunosuppressive TME (Supplementary Fig. 3D, http://links.lww.com/MD/J447).

To further explore the crosstalk between CAFs and tumor-associated macrophages (TAMs), we utilized the AUCell method, which revealed that CAFs represented an enriched activity in promoting macrophage differentiation into the M2 phenotype but not into the M1 phenotype (Fig. 6H).^[26]^ In previous studies, CAFs have been shown to secret CXCL12, C-C chemokine ligand 2 (CCL2), and endosialin (CD248), which can recruit monocytes and mediate M2 polarization of TAMs.^[15,41,42]^ The current study found that CXCL12, CCL2, and CD248 were mainly expressed in CAFs, indicating that CAFs could regulate TAM differentiation in PCa (Fig. 2A).

### 3.6. Prediction of responses to treatment

**Figure 7.**
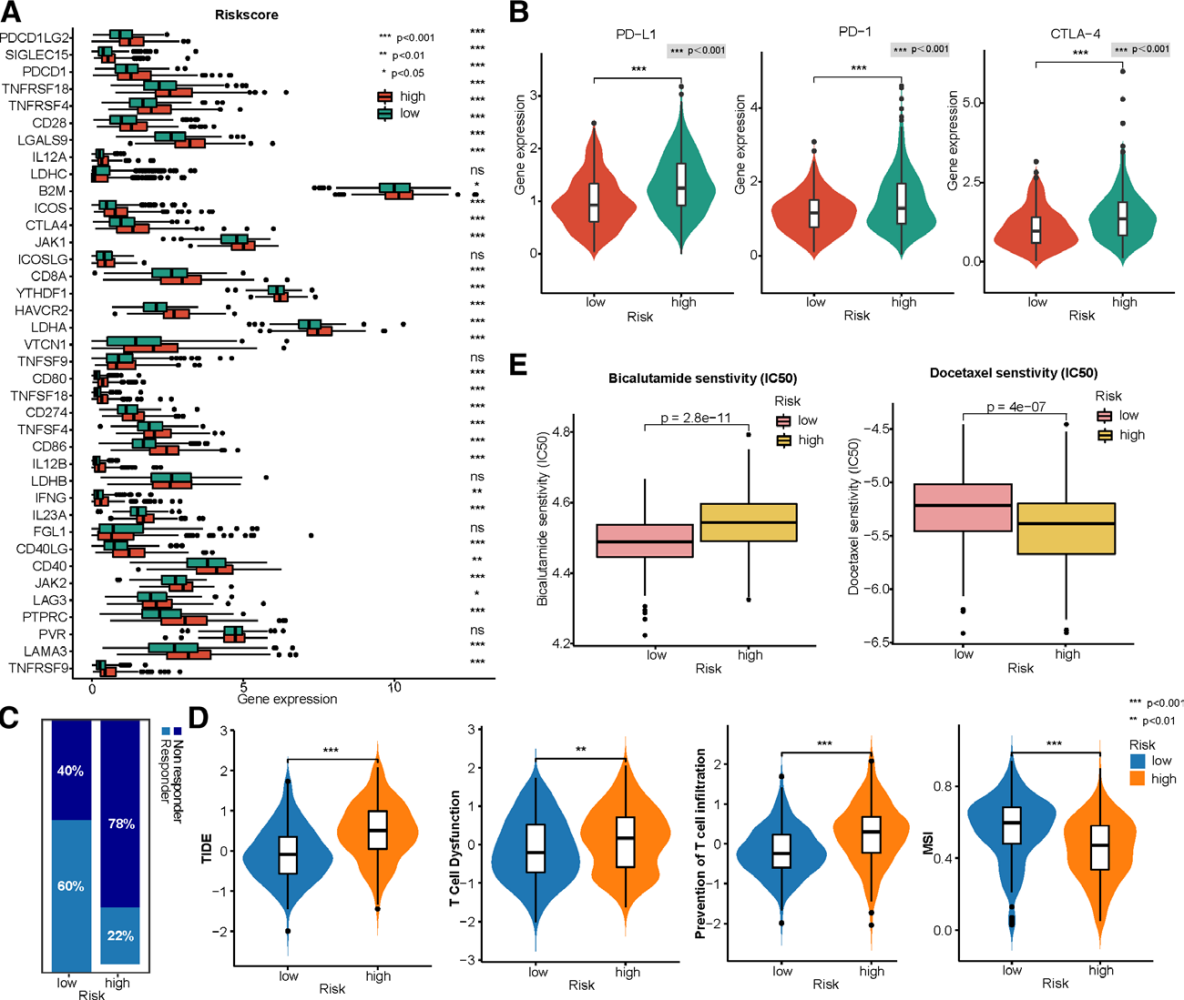
Prediction of the responses to treatment between 2 risk groups. (A–B) Differences in immune checkpoint molecules between high-risk and low-risk groups (A), especially focusing on the expression of PD-1, PD-L1, and CTLA-4 (B). (C) Proportion of the responder and non-responder to immunotherapy in 2 risk groups, which are defined by the Tumour Immune Dysfunction and Exclusion (TIDE) algorithm to assess the potentiality of tumor immune evasion. (D) Comparison of the scores of TIDE, T-cell dysfunction, the prevention of T cell infiltration, and microsatellite instability (MSI) between 2 risk groups. (E) Sensitivity to bicalutamide and docetaxel in 2 risk groups.

The crosstalk between CAFs and immune cells stimulates cancer progression and therapeutic resistance.^[15]^ In this study, we found that patients in the high-risk group were significantly associated with a higher expression of immune checkpoint molecules, such as PD-1, PD-L1, and CTLA4 (Fig. 7A–B), which are known to induce T cell dysfunction and predict poor prognosis.^[43]^ Blocking the immune checkpoint proteins via immunotherapy can help activate T cell function. However, a positive expression of PD-1/PD-L1 does not always indicate a good response to immune checkpoint inhibitor (ICI) therapy. Our results revealed that the high-risk group was significantly related to higher TIDE scores (Fig. 7C–D), indicating an elevated risk of tumor immunity escape and reduced reaction rate for immunotherapy in high-risk patients. CAFs, M2-TAMs, and myeloid-derived suppressor cells could arouse T cell dysfunction and prevent T cell infiltration into the tumor.^[15,33]^ Thus, we postulated that a combined treatment targeting CAFs might contribute to the effectiveness of immunotherapy in PCa. Additionally, our results showed that the low-risk group was sensitive to bicalutamide, a drug used in androgen deprivation therapy, and the high-risk group was sensitive to docetaxel (Fig. 7E).

### 3.7. Identification of CAF subpopulations in PCa

**Figure 8.**
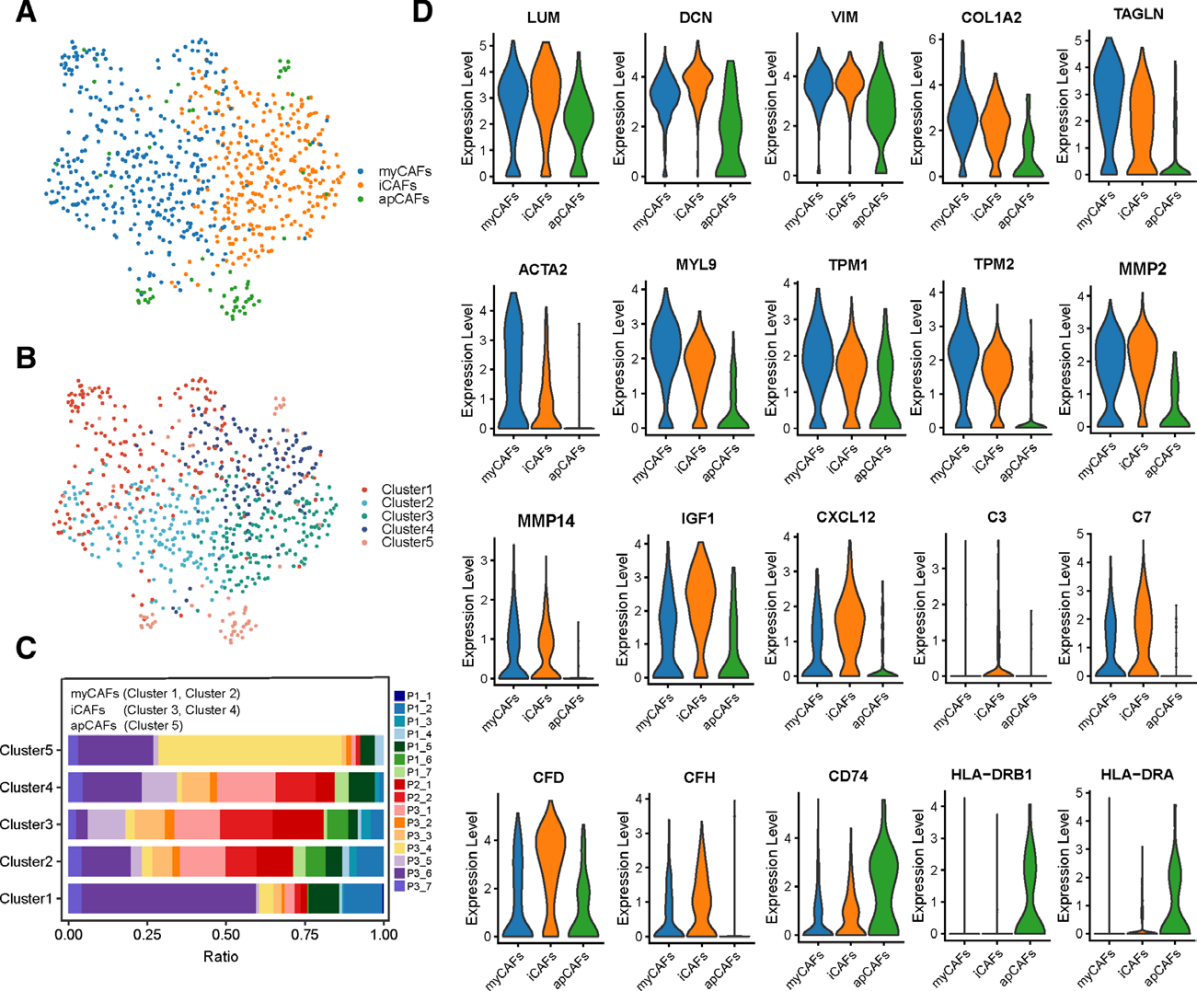
Three CAF subpopulations and their marker genes. (A–B) UMAP visualization of CAFs delineated by 3 CAFs subsets (A) and 5 clusters (B). (C) Proportion of 5 fibroblast clusters across 16 patients. (D) Violin plots demonstrate the distribution of canonical marker genes used to distinguish the CAFs subsets. CAFs = cancer-associated fibroblasts, UMAP = Uniform Manifold Approximation and Projection.

**Figure 9.**
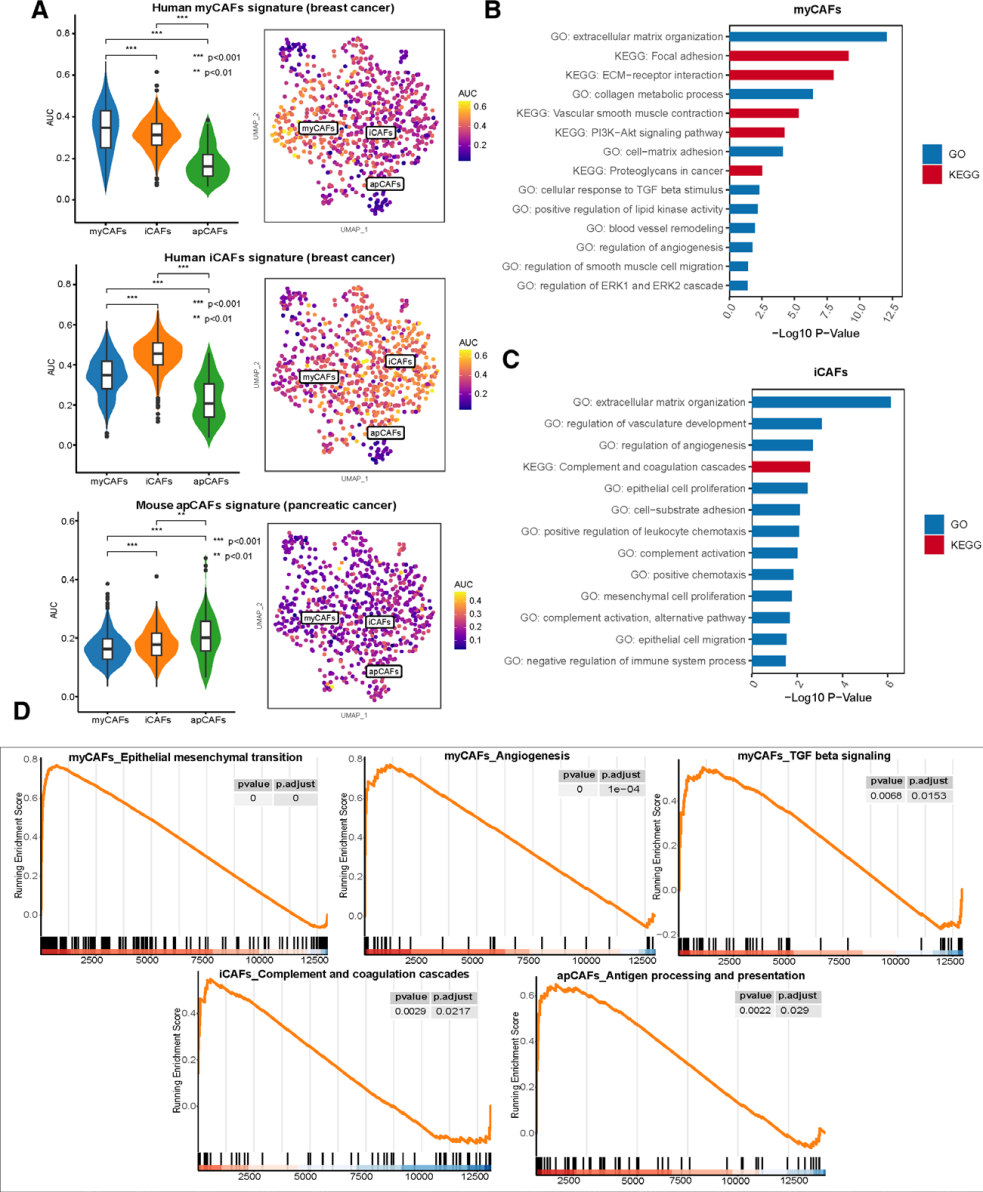
Validation of 3 CAF subsets according to the published CAF signatures and functional enrichment analyses. (A) AUC values for CAF subsets are scored based on the published CAF signatures in breast cancer (Elyada et al 2019) and pancreatic cancer (Wu et al 2020). Meanwhile, violin plots demonstrate the differences in AUC scores among 3 CAF subsets. (B–C) GO and KEGG analyses of the marker genes of myCAFs (B) and iCAFs (C). (D) The results of gene set enrichment analysis (GSEA) among 3 CAF subsets. AUC = area under the curve, CAFs = cancer-associated fibroblasts, iCAFs = inflammatory CAFs, GO = gene ontology, KEGG = Kyoto Encyclopedia of Genes and Genomes.

The present study integrated the scRNA-seq data of 16 PCa samples from 3 publicly available studies,^[16,18,19]^ thus increasing the number of CAFs in the scRNA-seq analysis of PCa. After re-clustering the CAFs via unsupervised clustering and Uniform Manifold Approximation and Projection, we identified 3 distinct CAF subpopulations with unique gene signatures, named myCAFs (myofibroblast-like CAFs), inflammatory CAFs (iCAFs) (immune and iCAFs), and apCAFs (Fig. 8A–C). DCN and LUM, the common fibroblast markers, and VIM, the marker gene of mesenchymal cells, were commonly expressed among the 3 CAF subsets, confirming the fibroblast annotation (Fig. 8D). MyCAFs highly expressed activated fibroblast markers (ACTA2 and TAGLN), ECM-associated genes (MYL9 and TPM), and collagen-related genes (COL1A1 and COL1A2).^[9]^ The canonical markers of iCAFs included inflammatory markers (CXCL12, IGF1) and complement-regulatory genes (C3, C7, CFD, and CFH).^[44]^ ApCAFs were characterized by the elevated expression of antigen-presenting genes (CD74, HLA-DRB1, and HLA-DRA) (Fig. 8D).^[12]^ In addition to the analysis of these marker genes, we performed AUCell analysis to confirm the classification of the CAFs in PCa using published CAF signatures.^[12,24]^ This revealed similar enrichment of myCAF, iCAF, and apCAF signatures from breast and pancreatic cancers in the 3 CAF clusters of PCs, suggesting similar CAF types across different cancers (Fig. 9A and Supplementary Fig. 5A, http://links.lww.com/MD/J449).

### 3.8. Functional assessment and pseudotime analysis of the CAF subpopulations

A total of 316 DEGs among the 3 CAF subsets were identified, and the expression of marker genes is shown in Supplementary Fig. 5B, http://links.lww.com/MD/J449 and Supplementary Table 3, http://links.lww.com/MD/J450. GO and KEGG analyses were conducted using these marker genes to reveal the pathway-level differences driving CAF heterogeneity. The upregulated markers in myCAFs were enriched in several classical myofibroblast-related processes, such as ECM remodeling, collagen metabolic process, cell adhesion, and smooth muscle contraction (Fig. 9B).^[9]^ In contrast, the results revealed the enrichment of complement activation, positive chemotaxis, and epithelial cell proliferation and migration in iCAFs (Fig. 9C). Besides, both myCAFs and iCAFs expressed high levels of the metalloproteinases MMP2 and MMP14, which are involved in ECM remodeling (Fig. 8D). GSEA also verified the enriched pathways in myCAFs (EMT, angiogenesis, transforming growth factor [TGF] β signaling), iCAFs (complement and coagulation cascades), and apCAFs (antigen processing and presentation) (Fig. 9D). Notably, the apCAF subset contained only 67 cells from 2 patients (Fig. 8C). A trajectory analysis conducted to determine the evolutionary property of the CAFs revealed that the iCAFs might be an initial subpopulation of the CAFs (Supplementary Fig. 6A–D, http://links.lww.com/MD/J451). Taken together, these results indicated that there were 3 CAF subtypes with unique functions in PCa.

## 4. Discussion

Even after undergoing radical treatment, patients with BCR in PCa may experience clinical progression within a median time of 5 to 8 years, leading to an increased risk of cancer-associated death.^[45]^ Therefore, identifying independent biomarkers for BCR is of great importance for the clinical treatment of PCa. Notably, considerable evidence has confirmed the impact of CAFs on tumor progression and metastasis.^[9]^ Thus, the present study, for the first time, constructed a CAF-derived prognostic signature to accurately predict patients with PCa at high risk of BCR in 5 independent PCa cohorts. The high-risk scores obtained from the CAF-related signature showed close associations with the infiltration of fibroblasts, stromal cells and immune cells, and especially positively related to macrophage M2 polarization. Meanwhile, the high-risk group demonstrated an increased probability of tumor immune escape, indicating that combined treatment targeting CAFs might contribute to the effectiveness of immunotherapy in PCa. Additionally, we identified 3 biologically distinct CAF subsets in PCa, namely myCAFs, iCAFs, and apCAFs.

CAFs act as a central cell-community role among different cells contributing to ECM remodeling, angiogenesis, cancer cell growth, and immune system regulation.^[5–7]^ Based on our analysis of cell-cell communication, we have discovered that CAFs engage in extensive communication with other cells. Undoubtedly, the present study verified the classic functions of CAFs in PCa via functional enrichment analysis. As demonstrated by our findings (Fig. 2A), ECM-modulating genes, such as MMP2 and MMP14, were clearly upregulated in the CAFs,^[46]^ and CAFs were found to secrete PDGFRA and CXCL12, which promote tumor progression,^[9]^ further validate the accuracy of CAF classification. Several scRNA-seq analyses have elucidated fibroblast heterogeneity in some cancer types.^[12,24,44]^ MyCAFs and iCAFs were revealed to have distinct morphologies and spatial distribution in breast cancer.^[24]^ In pancreatic ductal adenocarcinoma, Chen et al^[44]^ detected complement-secreting CAFs expressing high levels of C3, C7, CFD, and CFH, which might modulate the immune and inflammation response. Similarly, we identified myCAF and iCAF subsets in PCa and they were involved in unique biological processes, indicating that similar phenotypes likely exist across different tissues. Vickman et al^[17]^ cultured CAFs from primary PCa tissues and also discovered that a large cluster, characterized as inflammatory CAF subsets, was associated with myeloid cell recruitment. Interestingly, 1 CAF cluster in our study presented an enrichment for signatures of apCAFs, which were discovered in breast and pancreatic cancers.^[9,44]^ Nonetheless, the origin of the apCAF subset and its ability to mediate the activation of T cells remain poorly understood.^[9]^ Because of the limited number of apCAF cells in our study, more extensive studies will be required to assess the properties of apCAFs in PCa. Investigating the functions and heterogeneity of the CAFs can contribute to our understanding PCa pathology and aid in developing potential therapeutic strategies targeting CAFs.^[15]^

As we suspected, the CAF-related signature and its candidate genes were closely associated with the prognosis of the tumors. Several studies have developed prognostic signatures based on CAF marker genes in hepatocellular carcinoma,^[47]^ colorectal cancer,^[48]^ and lung cancer.^[49]^ Considering the similar CAF properties among different tumors, we developed a CAF-related prognostic signature in PCa for the first time. The CAF-related risk score was not only associated with higher levels of PSA, tumor stage, and GS but also displayed an independent correlation with the prognosis of PCa even after adjusting for these factors (OR 2.1, 95% CI 1.18–3.72). Furthermore, this signature predictive value has been validated across 5 independent datasets, consistently showing a high AUC, thereby further strengthening the evidence for its effectiveness in accurately predicting PCa prognosis. The signature consisted of 7 genes that have been verified to be associated with tumors. One of these genes is BGN, a signaling molecule derived from the ECM, which is involved in collagen fiber assembly, tumor invasion, and metastasis.^[50]^ Previous studies by Frank et al^[51]^ have demonstrated a positive correlation of BGN protein expression with prostate-specific antigen recurrence, TMPRRS2:ERG fusion, and PTEN deletion in PCa. AEBP1, another gene in the signature, plays a role in organizing and remodeling ECM and acts as a transcriptional repressor.^[52,53]^ AEBP1 overexpression can activate the NF-κB and PI3K-AKT pathways, thereby promoting tumor development in many malignancies, such as breast cancer, glioblastoma, and bladder cancer.^[52]^ Additionally, Robert et al^[54]^ demonstrated that ASPN, a novel factor of mesenchymal stromal cells and fibroblasts, could regulate the TME and promote metastatic progression. The expression of ASPN was significantly elevated in GS4 with cribriform morphology compared to that in GS4 with non-cribriform profile and GS3.^[55]^ Moreover, COL1A1 and ALDH1A1 have been identified as biomarkers associated with poor prognosis and promote tumor progression in distinct tumors,^[56,57]^ including PCa.^[58,59]^ As a protective gene, PAGE4 overexpression in stromal cells can attenuate androgen receptor activation to inhibit progression to advanced lethal PCa^[60]^ and decrease the migration and invasion of epithelial cells via TGF β and tumor necrosis factor-α pathways.^[61]^ Collectively, this CAF-derived gene signature could reliably predict the prognosis of BCR in patients with PCa.

The complicated crosstalk between CAFs and immune cells can lead to immunosuppression and stimulate T-cell dysfunction.^[15]^ We confirmed a positive association between the risk score of the CAF-related signature and the levels of fibroblasts and M2-TAMs. M2-TAMs are known to promote angiogenesis, immunosuppression, and tumor invasion and metastasis.^[62]^ Several studies have revealed that CAFs recruit monocytes to tumor cells and facilitate their differentiation into M2-TAMs through the CXCL12/CXCR4 pathway.^[41,63,64]^ Additionally, Yang et al^[42]^ reported that CAFs in hepatocellular carcinoma express high levels of endosialin (CD248), which interacted with CD68 to recruit monocytes and modulate GAS6 expression in CAFs, inducing M2 polarization. A similar expression of CXCL12, CD248, and GAS6 was observed in the present study, indicating that the CAFs in PCa might interact with TAMs through different pathways. ICI therapy shows limited anti-tumor activity in PCa,^[65,66]^ partly due to the poor infiltration of immune components in the TME.^[67]^ For example, Ford et al^[68]^ revealed that CAFs promoted the exclusion of CD8^+^ T cells from tumors and induced an immunosuppressive TME. In the current study, TIDE analysis revealed a higher probability of tumor immunity escape and prevention of T cell infiltration in the high-risk group. Additionally, CAFs can upregulate the expression of PD-1/PD-L1 and CTLA4/B7 in cancer cells and other cells within the TME to assist tumor cells in avoiding immune responses.^[15,69]^ Therefore, CAFs may play an important role in mediating resistance to ICI immunotherapy in PCa. In patients with a high-risk score, targeting CAFs might be a promising therapy to enhance the effectiveness of immunotherapy and synergistically counteract tumor progression.

Our study has several limitations. First, it was difficult to define fibroblasts due to a lack of unique markers that are not expressed in other cells. We identified fibroblasts according to the marker genes used in the published scRNA-seq analysis.^[18,38,39]^ Second, the number of patients included in this study was small. Further studies are needed to validate the complexity of the CAFs. Third, although we verified 3 CAFs subpopulations using GSEA, functional enrichment analysis, and AUCell score, the spatial distribution and their specific functions were not further explored.

## 5. Conclusion

In summary, we established and validated a CAF-derived prognostic signature by combining single-cell and bulk RNA sequencing, which can accurately predict the biochemical relapse of PCa and reflect the levels of immunosuppression in the TME. Furthermore, 3 biologically distinct CAF subpopulations were identified in PCa. Understanding the functions and heterogeneity of CAFs could provide potential therapeutic strategies for PCa.

## Acknowledgments

We want to acknowledge the participants and researchers of the TCGA, MSKCC, GSE141445, GSE157703, GSE185344, GSE54460, GSE116918, and GSE46602 for providing the publicly available data used in this study. This research was funded by National High Level Hospital Clinical Research Funding, grant number BJ-2018-090, and National High Level Hospital Clinical Research Funding, grant number BJ-2022-157.

## Author contributions

**Conceptualization:** Wen Liu, Ming Liu.

**Data curation:** Wen Liu, Miaomiao Wang, Miao Wang.

**Methodology:** Wen Liu, Miaomiao Wang, Ming Liu.

**Software:** Wen Liu, Miao Wang.

**Validation:** Wen Liu, Miaomiao Wang.

**Writing – original draft:** Wen Liu, Miaomiao Wang.

**Writing – review & editing:** Miao Wang, Ming Liu.

## Supplementary Material


















