# Supplementary material for: Single-cell and bulk RNA sequencing reveal cancer-associated fibroblast heterogeneity and a prognostic signature in prostate cancer
Source: Medicine (Baltimore). 2023 Aug 11;102(32):e34611. doi: 10.1097/MD.0000000000034611 (PMC10419654; doi:10.1097/MD.0000000000034611)
Supplement: Supplementary file 1 [file medi-102-e34611-s001.pdf]

Supplementary Figure 1. Quality control and single-cell gene expression profiling. (a) The results of quality control, including the number of RNA features (nFeature_RNA), absolute UMI counts (nCount_RNA), and the percent of mitochondrial genes (percent.mt); (b) Correlation analysis between different features; (c-d) UMAP visualization is delineated by clusters (c) and number of features (d); (e) Log-normalised expression of markers for epithelial cells (KRT18), endothelial cells (PECAM1), myeloid cells (MS4A6A), smooth muscle cell (RGS5), T cells (CD3D), mast cells (TPSAB1), fibroblasts (LUM), B cells (MS4A1), and proliferative cells (MKI67).

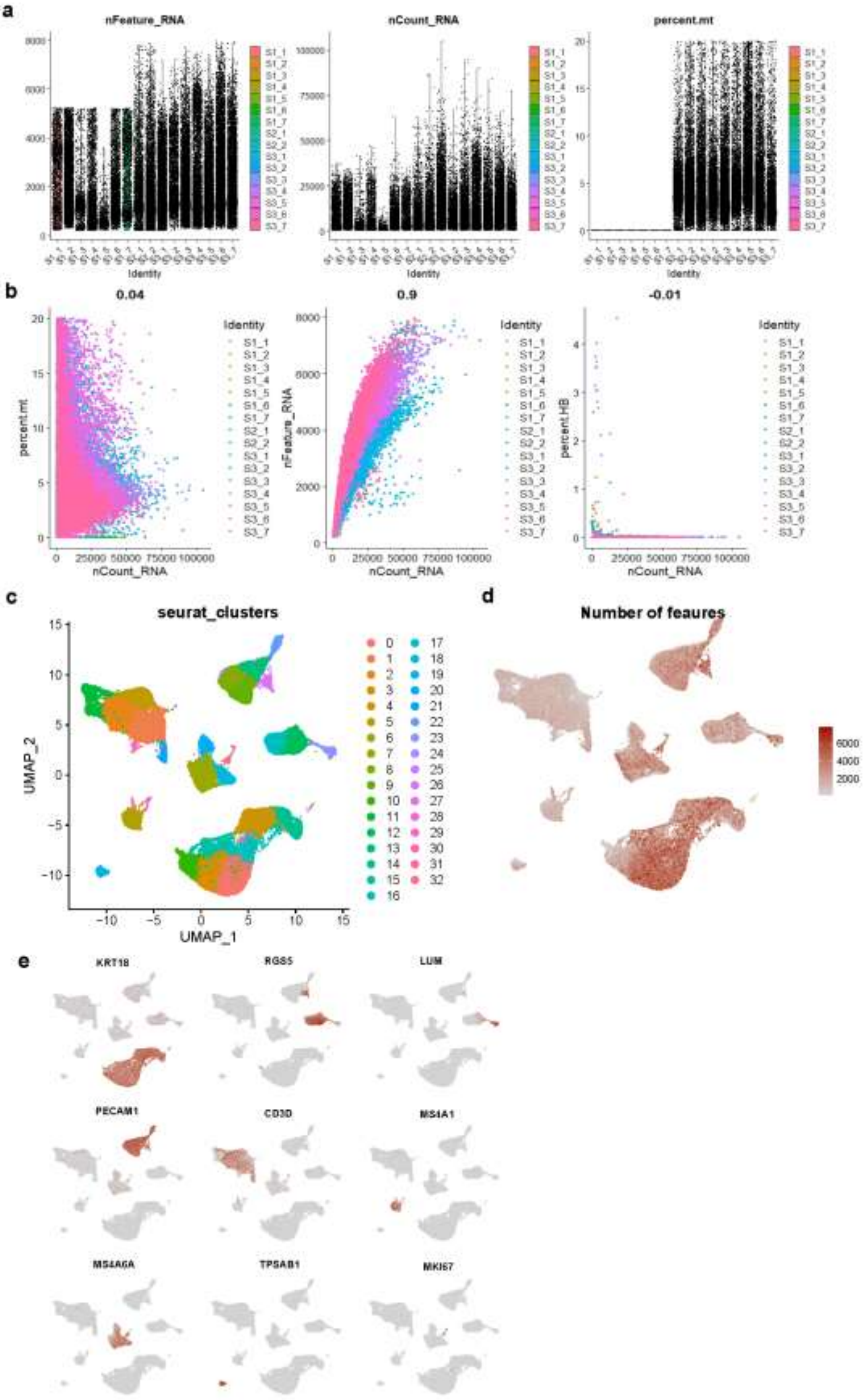
a
nFeature_RNA
nCount_RNA
percent.mt
Identity
S1_1
S1_2
S1_3
S1_4
S1_5
S1_6
S1_7
S2_1
S2_2
S3_1
S3_2
S3_3
S3_4
S3_5
S3_6
S3_7
b
0.04
0.9
-0.01
percent.mt
nFeature_RNA
percent.HB
nCount_RNA
c
seurat_clusters
UMAP_1
UMAP_2
d
Number of feaures
e
KRT18
RGS5
LUM
PECAM1
CD3D
MS4A1
MS4A6A
TPSAB1
MKI67
