# Supplementary material for: Single-cell and bulk RNA sequencing reveal cancer-associated fibroblast heterogeneity and a prognostic signature in prostate cancer
Source: Medicine (Baltimore). 2023 Aug 11;102(32):e34611. doi: 10.1097/MD.0000000000034611 (PMC10419654; doi:10.1097/MD.0000000000034611)
Supplement: Supplementary file 2 [file medi-102-e34611-s002.pdf]

Supplementary Table 1. The marker genes of cancer-associated fibroblasts (CAFs) based on the single-cell RNA sequencing analysis.

gene	avg_log2FC	gene	avg_log2FC	gene	avg_log2FC
APOD	6.58	OLFML3	1.81	RASD1	1.34
PTGDS	5.03	TIMP2	1.79	CFH	1.34
DCN	4.97	MATN2	1.78	PDGFRA	1.32
FBLN1	4.79	COL8A1	1.78	FXYD1	1.31
LUM	4.75	MFGE8	1.75	PEMT	1.3
CFD	4.12	DPT	1.75	SELENOM	1.3
COL1A2	3.87	PODN	1.74	TUBA1A	1.29
MGP	3.84	VCAN	1.73	HSD11B1	1.29
IGFBP5	3.67	NR2F1	1.73	ASPN	1.28
COL1A1	3.63	GEM	1.72	PMP22	1.28
SERPINF1	3.44	PLAC9	1.72	MYL9	1.27
IGFBP6	3.33	CXCL12	1.72	CCL2	1.26
TIMP1	3.32	FBLN2	1.71	RAB34	1.26
COL3A1	3.18	DIO2	1.7	VIM	1.26
BGN	3.15	TPM2	1.69	SRPX	1.23
PTN	3.14	FXYD6	1.68	ANXA5	1.22
SFRP4	3.12	SSPN	1.67	S100A13	1.22
COL6A2	3.1	EMILIN1	1.66	HTRA3	1.19
LTBP4	3.02	ABCA8	1.65	CD63	1.19
CCDC80	2.99	LRP1	1.65	FOXF1	1.18
SFRP2	2.97	GPM6B	1.64	NR2F2	1.17
MFAP4	2.94	OGN	1.63	OAF	1.17
CCN2	2.93	EFEMP1	1.63	TNC	1.17
IGF1	2.91	SPARCL1	1.63	MXRA7	1.15
MMP2	2.89	ACTA2	1.6	CNN3	1.15
GSN	2.86	CYGB	1.6	IFITM3	1.14
C1S	2.84	RARRES1	1.58	SBDS	1.13
RARRES2	2.76	AKAP12	1.57	MAP1B	1.13
SFRP1	2.76	SPOCK3	1.56	NFIC	1.13
LGALS1	2.6	CSRP1	1.55	CRTAP	1.13
MXRA8	2.5	PRRX1	1.54	FGF7	1.12
PCOLCE	2.48	THBS1	1.53	CYBRD1	1.12
PAGE4	2.48	ALDH1A1	1.53	HTRA1	1.12
C11ORF96	2.47	MOXD1	1.52	CD81	1.12
TCF21	2.46	ELN	1.51	PID1	1.12
SERPING1	2.43	S100A6	1.51	PLD3	1.11
COL6A1	2.34	PCOLCE2	1.5	PDGFRB	1.11
FN1	2.28	PRELP	1.49	TFPI	1.11
C1R	2.28	TIMP3	1.49	EID1	1.1
DKK3	2.26	GAS6	1.48	CEBPD	1.09
NBL1	2.25	PALLD	1.47	TCEAL4	1.09

COL6A3	2.25	CTHRC1	1.47	COL4A2	1.08
ISLR	2.22	A2M	1.47	EPHA3	1.08
C7	2.22	FSTL1	1.46	MMP14	1.06
IGFBP2	2.2	ECM1	1.45	RCN3	1.06
TAGLN	2.19	SERPINE2	1.44	LGALS3	1.06
IGFBP4	2.18	COL14A1	1.43	CD248	1.05
SERPINE1	2.17	FHL2	1.43	RORB	1.04
SPARC	2.11	CTGF	1.42	CTSF	1.04
AEBP1	2.1	MFAP2	1.41	MRC2	1.04
SOD3	2.08	COL18A1	1.39	RND3	1.04
HSPB6	2.05	PAMR1	1.39	PKIG	1.03
CYP1B1	2.04	GPC3	1.38	ID3	1.03
PLTP	2.02	CYR61	1.37	DDR2	1.03
CTSK	2.01	TPM1	1.37	RDH10	1.03
IGFBP7	2	FBLN5	1.36	CYB5R3	1.03
CALD1	1.99	THY1	1.36	SCN7A	1.03
CRISPLD2	1.92	LAPTM4A	1.35	COL5A1	1.02
LGALS3BP	1.88	GGT5	1.35	CDH11	1.01
MEG3	1.88	EFEMP2	1.35	PDLIM7	1.01
PLPP3	1.82	SULF1	1.34	EDNRA	1

Abbreviation: FC, fold change.
