# Supplementary material for: Single-cell and bulk RNA sequencing reveal cancer-associated fibroblast heterogeneity and a prognostic signature in prostate cancer
Source: Medicine (Baltimore). 2023 Aug 11;102(32):e34611. doi: 10.1097/MD.0000000000034611 (PMC10419654; doi:10.1097/MD.0000000000034611)

Supplementary Figure 2. Cell communication analysis. (a-b) Cross-talk analysis between each cell types in PCa. The thickness of the line indicates the number (a) or interaction weights (b) of ligand-receptor pairs; (c) Bubble plot shows ligand-receptor pairs between fibroblasts and other cells, including epithelial, endothelial, and smooth muscle cells.

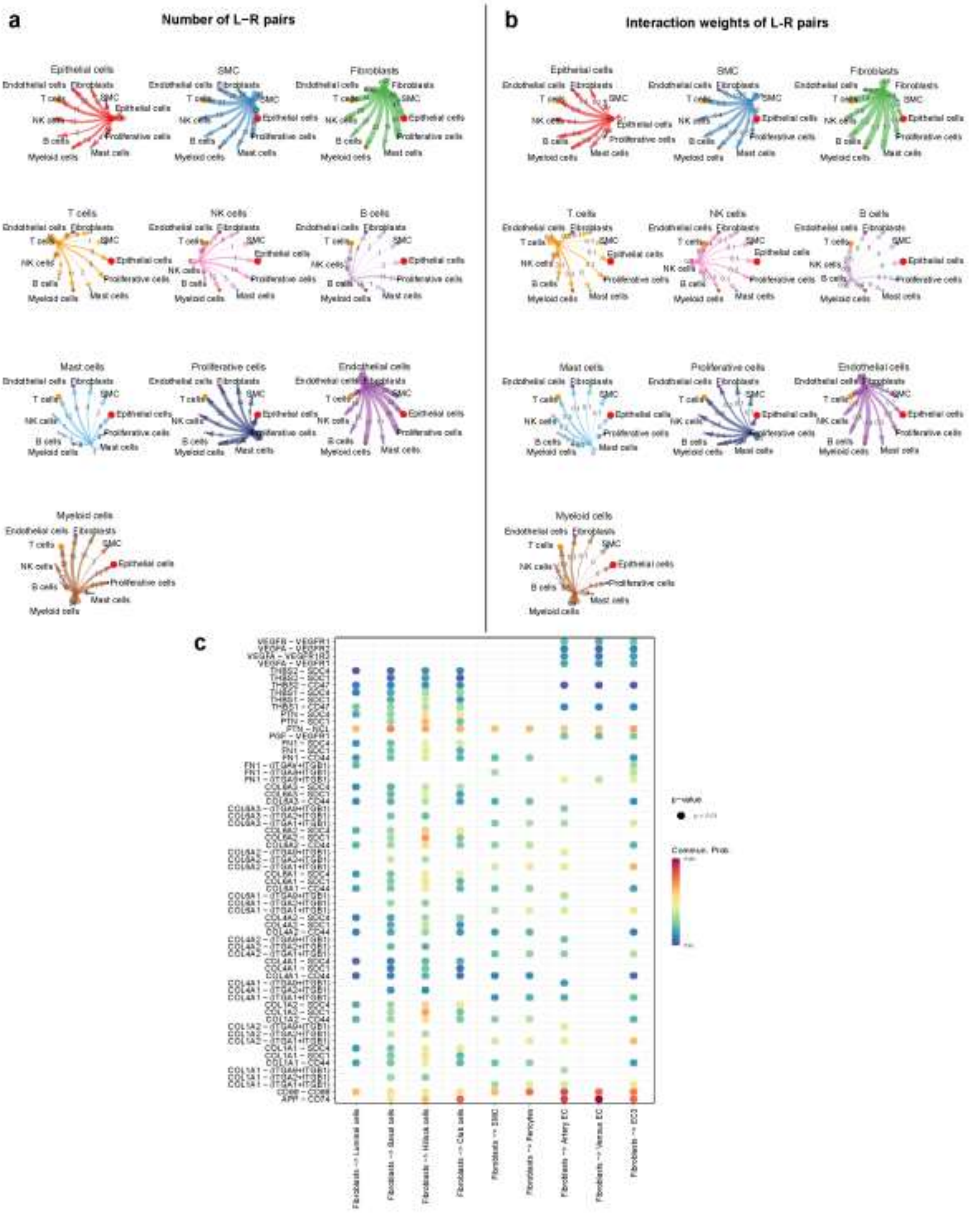

Supplement: Supplementary file 3 [file medi-102-e34611-s003.pdf]
