# Supplementary material for: Single-cell and bulk RNA sequencing reveal cancer-associated fibroblast heterogeneity and a prognostic signature in prostate cancer
Source: Medicine (Baltimore). 2023 Aug 11;102(32):e34611. doi: 10.1097/MD.0000000000034611 (PMC10419654; doi:10.1097/MD.0000000000034611)
Supplement: Supplementary file 4 [file medi-102-e34611-s004.pdf]

Supplementary Table 2. Univariable Cox regression analysis identified CAFs-related marker genes associated with the biochemical relapse-free survival (bRFS) in the TCGA cohort.

Gene	HR (95%CIs)	Pvalue	Gene	HR (95%CIs)	Pvalue
COL1A2	1.47 (1.17, 1.85)	0.001	MOXD1	1.32 (1.06, 1.63)	0.013
MGP	1.59 (1.25, 2.02)	<0.001	ELN	1.44 (1.15, 1.8)	0.001
COL1A1	1.59 (1.3, 1.95)	<0.001	PRELP	1.4 (1.09, 1.79)	0.008
COL3A1	1.41 (1.15, 1.73)	0.001	GAS6	1.35 (1.01, 1.82)	0.043
BGN	1.85 (1.44, 2.36)	<0.001	CTHRC1	1.54 (1.27, 1.86)	<0.001
SFRP4	1.34 (1.15, 1.58)	<0.001	SERPINE2	1.31 (1.02, 1.7)	0.037
SFRP2	1.4 (1.15, 1.69)	0.001	MFAP2	1.59 (1.18, 2.16)	0.003
MFAP4	1.37 (1.09, 1.72)	0.007	COL18A1	1.44 (1.05, 1.99)	0.024
MXRA8	1.89 (1.37, 2.61)	<0.001	FBLN5	1.36 (1.05, 1.76)	0.022
PAGE4	0.83 (0.72, 0.96)	0.01	THY1	1.63 (1.25, 2.13)	<0.001
FN1	1.18 (1.01, 1.38)	0.033	GGT5	1.37 (1.02, 1.84)	0.039
C1R	1.29 (1.01, 1.64)	0.038	SULF1	1.39 (1.13, 1.71)	0.002
ISLR	1.38 (1.06, 1.81)	0.017	RASD1	0.82 (0.68, 0.98)	0.028
SPARC	1.56 (1.21, 2)	0.001	ASPN	1.58 (1.3, 1.93)	<0.001
AEBP1	1.75 (1.36, 2.25)	<0.001	VIM	1.39 (1.02, 1.88)	0.035
CTSK	1.55 (1.16, 2.07)	0.003	OAF	1.72 (1.19, 2.47)	0.003
IGFBP7	1.62 (1.21, 2.17)	0.001	HTRA1	1.43 (1.01, 2.02)	0.042
TIMP2	1.34 (1.01, 1.77)	0.045	PDGFRB	1.36 (1.06, 1.76)	0.017
COL8A1	1.47 (1.2, 1.8)	<0.001	COL4A2	1.38 (1.06, 1.79)	0.018
MFGE8	1.56 (1.17, 2.08)	0.003	RCN3	1.69 (1.27, 2.24)	<0.001
VCAN	1.4 (1.16, 1.68)	<0.001	PKIG	1.56 (1.06, 2.31)	0.025
DIO2	1.27 (1.01, 1.58)	0.037	COL5A1	1.53 (1.19, 1.95)	0.001
ALDH1A1	1.22 (1.07, 1.4)	0.003	CDH11	1.45 (1.13, 1.86)	0.003

Abbreviation: HR, hazard ratio; CI, confidence interval; CAF, cancer-associated fibroblast
