# Supplementary material for: Single-cell and bulk RNA sequencing reveal cancer-associated fibroblast heterogeneity and a prognostic signature in prostate cancer
Source: Medicine (Baltimore). 2023 Aug 11;102(32):e34611. doi: 10.1097/MD.0000000000034611 (PMC10419654; doi:10.1097/MD.0000000000034611)
Supplement: Supplementary file 5 [file medi-102-e34611-s005.pdf]

Supplementary Figure 3. KM survival curves and a prognostic nomogram. (a) KM survival curves of biochemical relapse-free survival (bRFS) in seven candidate genes; (b) A prognostic nomogram including risk score and other clinical factors; (c) The calibration curves of the 1-, 3-, and 5-year bRFS; (d) The correlation between 22 immune cells and fibroblasts. The fibroblast signature score was calculated by GSVA based on all fibroblast marker genes.

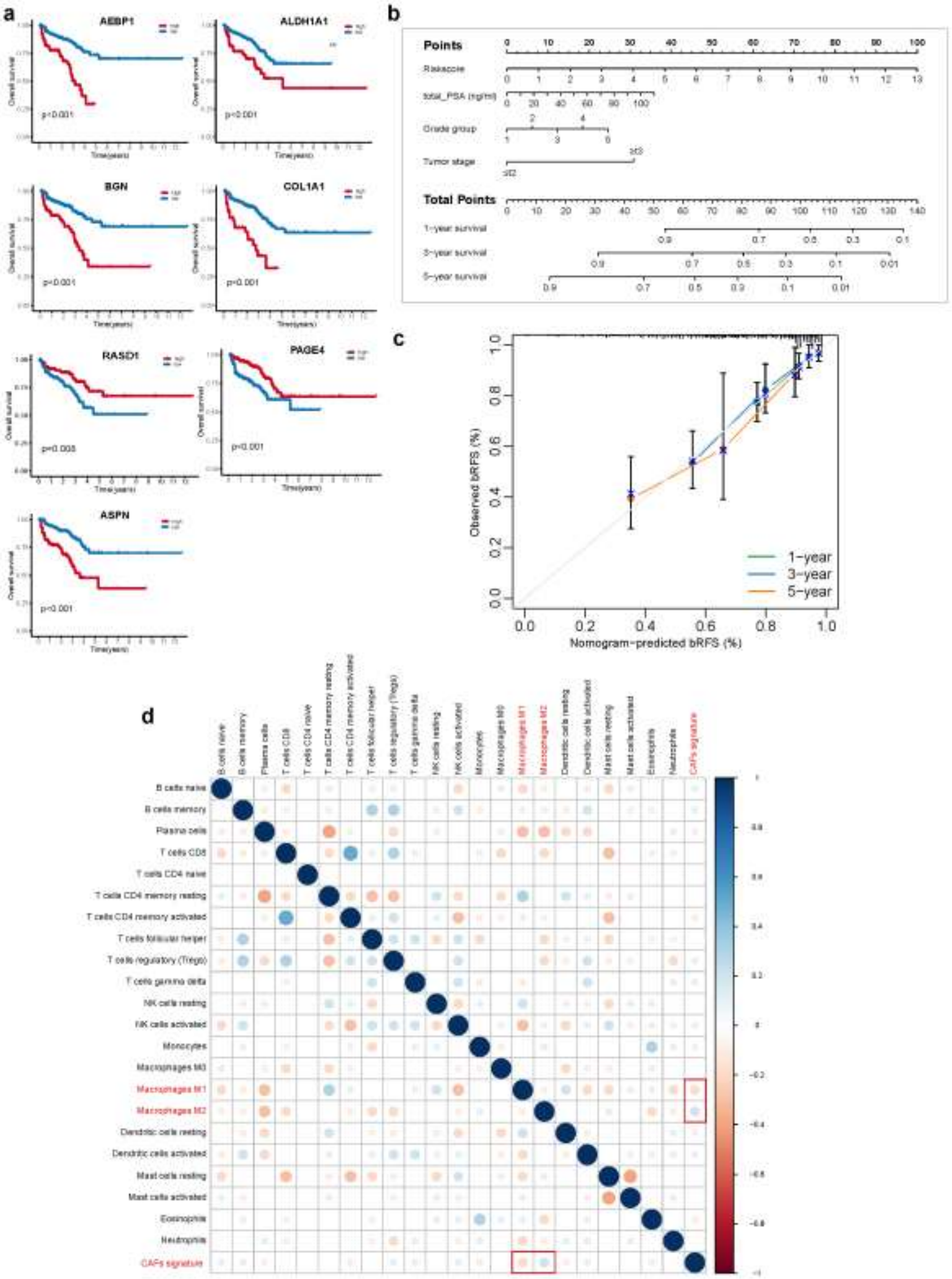
a
AEBP1
p<0.001
ALDH1A1
p<0.001
BGN
p<0.001
COL1A1
p<0.001
RASD1
p=0.008
PAGE4
p<0.001
ASPN
p<0.001
Overall survival
Time(years)
b
Points
0
10
20
30
40
50
60
70
80
90
100
Riskscore
0
1
2
3
4
5
6
7
8
9
10
11
12
13
total_PSA (ng/ml)
0
20
40
60
80
100
Grade group
1
2
3
4
5
Tumor stage
≤t2
≥t3
Total Points
0
10
20
30
40
50
60
70
80
90
100
110
120
130
140
1-year survival
0.9
0.7
0.5
0.3
0.1
3-year survival
0.9
0.7
0.5
0.3
0.1
0.01
5-year survival
0.9
0.7
0.5
0.3
0.1
0.01
c
Observed bRFS (%)
Nomogram-predicted bRFS (%)
0.0
0.2
0.4
0.6
0.8
1.0
1-year
3-year
5-year
d
B cells naive
B cells memory
Plasma cells
T cells CD8
T cells CD4 naive
T cells CD4 memory resting
T cells CD4 memory activated
T cells follicular helper
T cells regulatory (Tregs)
T cells gamma delta
NK cells resting
NK cells activated
Monocytes
Macrophages M0
Macrophages M1
Macrophages M2
Dendritic cells resting
Dendritic cells activated
Mast cells resting
Mast cells activated
Eosinophils
Neutrophils
CAFs signature
