# Supplementary material for: Single-cell and bulk RNA sequencing reveal cancer-associated fibroblast heterogeneity and a prognostic signature in prostate cancer
Source: Medicine (Baltimore). 2023 Aug 11;102(32):e34611. doi: 10.1097/MD.0000000000034611 (PMC10419654; doi:10.1097/MD.0000000000034611)

Supplementary Figure 4. Immunohistochemical (IHC) and immunofluorescent (IF) staining present the protein expressions of candidate genes based on samples from Beijing hospital (a-d) and the Human Protein Atlas (HPA) database (e-k).

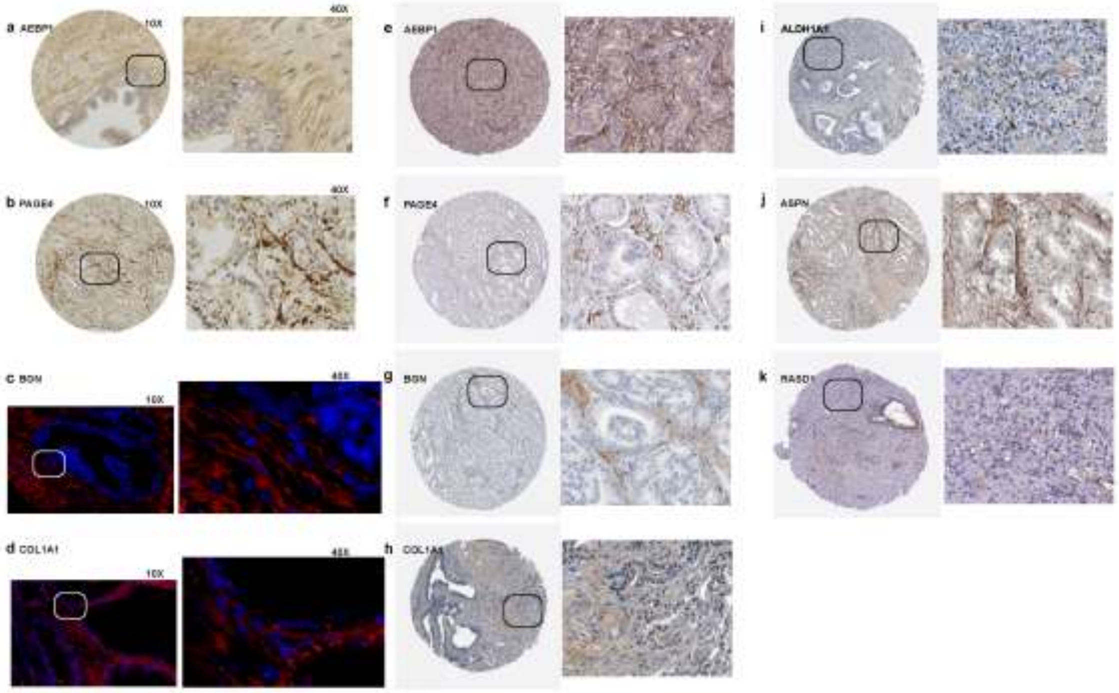

Supplement: Supplementary file 6 [file medi-102-e34611-s006.pdf]
