# Supplementary material for: Single-cell and bulk RNA sequencing reveal cancer-associated fibroblast heterogeneity and a prognostic signature in prostate cancer
Source: Medicine (Baltimore). 2023 Aug 11;102(32):e34611. doi: 10.1097/MD.0000000000034611 (PMC10419654; doi:10.1097/MD.0000000000034611)

Supplementary Figure 5. AUC scores and heatmap. (a) AUC values in three CAF subsets are scored based on the published CAF signatures in pancreatic cancer (Wu et al. 2020). Meanwhile, violin plots show the differences in AUC scores among three CAF subsets; (b) Heatmap presents the top marker genes in three CAF subsets.

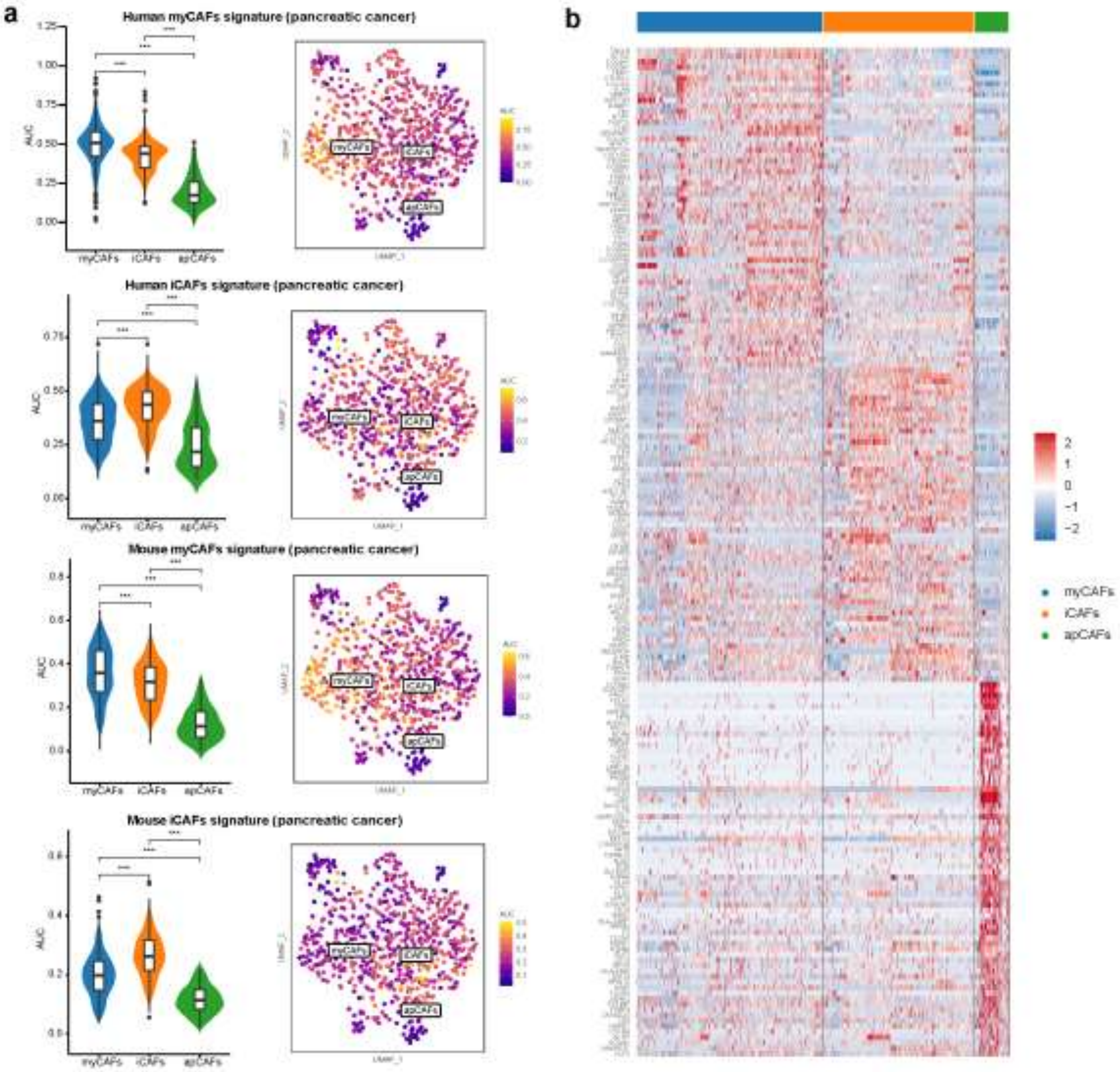

Supplement: Supplementary file 7 [file medi-102-e34611-s007.pdf]
