# Supplementary material for: Single-cell and bulk RNA sequencing reveal cancer-associated fibroblast heterogeneity and a prognostic signature in prostate cancer
Source: Medicine (Baltimore). 2023 Aug 11;102(32):e34611. doi: 10.1097/MD.0000000000034611 (PMC10419654; doi:10.1097/MD.0000000000034611)
Supplement: Supplementary file 8 [file medi-102-e34611-s008.pdf]

Supplementary Table 3. The marker genes of three CAF subsets based on the single-cell RNA sequencing analysis.

Cluster	gene	avg_log2FC	Cluster	gene	avg_log2FC	Cluster	gene	avg_log2FC
myCAFs	ACTA2	1.83	iCAFs	PGRMC1	0.56	iCAFs	TMEM45A	0.3
myCAFs	COL3A1	1.53	iCAFs	RP11-452	0.55	iCAFs	OLFML1	0.3
myCAFs	COL1A1	1.53	iCAFs	CES1	0.54	iCAFs	PIGT	0.3
myCAFs	TAGLN	1.39	iCAFs	RGS2	0.54	iCAFs	RILPL2	0.3
myCAFs	CCN2	1.38	iCAFs	FCGRT	0.54	iCAFs	IP6K2	0.3
myCAFs	COL4A1	1.22	iCAFs	GPNMB	0.53	iCAFs	FGF7	0.3
myCAFs	MYH11	1.19	iCAFs	CPVL	0.52	iCAFs	SLC22A17	0.29
myCAFs	FN1	1.12	iCAFs	GSN	0.52	iCAFs	PLBD1	0.29
myCAFs	SERPINE1	1.12	iCAFs	FOXF1	0.52	iCAFs	CETN2	0.29
myCAFs	COL1A2	1.1	iCAFs	FAIM2	0.52	iCAFs	RHOBTB3	0.29
myCAFs	COL4A2	1.05	iCAFs	GPX3	0.51	iCAFs	VAMP5	0.29
myCAFs	RAMP1	1.04	iCAFs	DKK3	0.51	iCAFs	NDUFS4	0.29
myCAFs	SPARC	1.04	iCAFs	PRSS23	0.5	iCAFs	LINC01082	0.29
myCAFs	CTHRC1	1.02	iCAFs	RPRM	0.49	iCAFs	FBXO17	0.29
myCAFs	TIMP1	1.01	iCAFs	SPARCL1	0.49	iCAFs	GABARAPL2	0.28
myCAFs	MYL9	1	iCAFs	HSD11B1	0.48	iCAFs	AK3	0.28
myCAFs	VCAN	1	iCAFs	SMOC1	0.47	iCAFs	CHRDL1	0.28
myCAFs	THBS4	0.93	iCAFs	CTSK	0.47	iCAFs	ARL6IP5	0.28
myCAFs	TPM2	0.92	iCAFs	C1S	0.47	iCAFs	MITF	0.27
myCAFs	BGN	0.86	iCAFs	MATN2	0.46	iCAFs	FBLN2	0.27
myCAFs	NOTCH3	0.85	iCAFs	BDH2	0.46	iCAFs	SDHD	0.27
myCAFs	COL5A1	0.84	iCAFs	PGF	0.46	iCAFs	ANXA4	0.27
myCAFs	COL8A1	0.84	iCAFs	SH3BP5	0.46	iCAFs	OAT	0.27
myCAFs	FLNA	0.8	iCAFs	RNASE4	0.45	iCAFs	LINC00493	0.27
myCAFs	CD81	0.79	iCAFs	SEPP1	0.45	iCAFs	HACD3	0.26
myCAFs	ITGBL1	0.78	iCAFs	C14orf2	0.45	iCAFs	RPA2	0.26
myCAFs	FILIP1L	0.76	iCAFs	CTSH	0.45	iCAFs	MAMDC2	0.26
myCAFs	MYLK	0.75	iCAFs	LAPTM4A	0.44	iCAFs	PARVB	0.26
myCAFs	THBS2	0.75	iCAFs	CTSF	0.44	iCAFs	PRRX1	0.25
myCAFs	TPM1	0.73	iCAFs	SPG20	0.44	iCAFs	CD59	0.25
myCAFs	ITGB1	0.73	iCAFs	MMP2	0.44	apCAFs	KLK2	3.59
myCAFs	TNFRSF12A	0.71	iCAFs	SERPING1	0.44	apCAFs	NPY	3.2
myCAFs	PDLIM3	0.7	iCAFs	GLUL	0.44	apCAFs	PRAC1	3.01
myCAFs	MXRA7	0.69	iCAFs	NDNF	0.43	apCAFs	SCHLAP1	2.78
myCAFs	ENC1	0.68	iCAFs	CFH	0.43	apCAFs	KLK3	2.43
myCAFs	HOPX	0.67	iCAFs	GYPC	0.43	apCAFs	CD69	2.39
myCAFs	MYH9	0.66	iCAFs	MDK	0.43	apCAFs	COL9A2	2.39
myCAFs	CALD1	0.66	iCAFs	FKBP9	0.42	apCAFs	FOLH1	2.19
myCAFs	CSRP1	0.64	iCAFs	GAS6	0.42	apCAFs	SNHG29	1.94
myCAFs	PALLD	0.64	iCAFs	PRNP	0.42	apCAFs	TRGC1	1.88
myCAFs	TGFB1	0.64	iCAFs	MT-ND4	0.41	apCAFs	CD74	1.86
myCAFs	ITGA1	0.59	iCAFs	ADD3	0.41	apCAFs	BCAM	1.84

myCAFs	COL18A1	0.54	iCAFs	SOCS2	0.41	apCAFs	MARCKSL1	1.76
myCAFs	PDGFRB	0.54	iCAFs	ALDH1A2	0.41	apCAFs	HLA-DRA	1.75
myCAFs	COL12A1	0.53	iCAFs	TMCO1	0.41	apCAFs	TACSTD2	1.75
myCAFs	TPM4	0.53	iCAFs	AKR1C1	0.41	apCAFs	KRT18	1.73
myCAFs	COL16A1	0.53	iCAFs	GSTM5	0.41	apCAFs	AMACR	1.71
myCAFs	IGFBP7	0.52	iCAFs	PSAP	0.4	apCAFs	HLA-DRB1	1.68
myCAFs	UACA	0.52	iCAFs	ADM	0.4	apCAFs	C19ORF48	1.65
myCAFs	ANTXR1	0.5	iCAFs	HSPB6	0.4	apCAFs	ERG	1.6
myCAFs	CTSC	0.5	iCAFs	TCN2	0.39	apCAFs	HSPA6	1.59
myCAFs	PMEPA1	0.5	iCAFs	MTRNR2L1	0.39	apCAFs	KRT8	1.57
myCAFs	NPTN	0.47	iCAFs	FTL	0.39	apCAFs	HPN	1.52
myCAFs	FHL3	0.47	iCAFs	SRD5A2	0.39	apCAFs	SPDEF	1.52
myCAFs	FAM20C	0.47	iCAFs	LDHB	0.38	apCAFs	EEF1G	1.51
myCAFs	CNN3	0.44	iCAFs	GLT8D2	0.38	apCAFs	SYNGR2	1.51
myCAFs	PABPN1	0.42	iCAFs	PLD3	0.38	apCAFs	CLSTN2	1.51
myCAFs	CDK2AP1	0.42	iCAFs	SNAI2	0.38	apCAFs	OR51E1	1.5
myCAFs	FAP	0.4	iCAFs	C1orf21	0.38	apCAFs	LRRC26	1.5
myCAFs	MYL6	0.39	iCAFs	NBL1	0.37	apCAFs	TRPM4	1.47
myCAFs	PPP1R14B	0.39	iCAFs	UBB	0.37	apCAFs	F3	1.47
myCAFs	MIR4435-2HG	0.39	iCAFs	LRPAP1	0.37	apCAFs	KLK4	1.46
myCAFs	LINC01578	0.35	iCAFs	CIRBP	0.37	apCAFs	HOXB13	1.44
myCAFs	ALYREF	0.31	iCAFs	OMD	0.36	apCAFs	HSPE1	1.38
myCAFs	GXYLT2	0.3	iCAFs	ITM2B	0.36	apCAFs	KLK11	1.37
iCAFs	CFD	1.64	iCAFs	TMEM100	0.36	apCAFs	PPFIA2	1.36
iCAFs	IGF1	1.26	iCAFs	PODN	0.36	apCAFs	AGR2	1.36
iCAFs	DPT	1.18	iCAFs	MMP23B	0.36	apCAFs	ATP5MC2	1.35
iCAFs	CLU	1.18	iCAFs	TCEAL8	0.36	apCAFs	SRGN	1.35
iCAFs	PAGE4	1.13	iCAFs	RAI2	0.36	apCAFs	TSPAN13	1.34
iCAFs	PTN	1.08	iCAFs	PRCP	0.36	apCAFs	CLDN3	1.33
iCAFs	FBLN1	1.02	iCAFs	ADIRF	0.36	apCAFs	SFN	1.32
iCAFs	PCOLCE2	1	iCAFs	TSPAN3	0.36	apCAFs	TSTD1	1.32
iCAFs	CXCL12	0.98	iCAFs	ALDH1A3	0.36	apCAFs	LINC00910	1.3
iCAFs	SERPINF1	0.95	iCAFs	CYB5R3	0.35	apCAFs	SMIM22	1.26
iCAFs	PLPP3	0.94	iCAFs	COL14A1	0.35	apCAFs	IFI30	1.24
iCAFs	SRPX	0.93	iCAFs	LGALS3	0.35	apCAFs	MLPH	1.18
iCAFs	COCH	0.91	iCAFs	GSTM3	0.35	apCAFs	EPCAM	1.18
iCAFs	DCN	0.87	iCAFs	PID1	0.35	apCAFs	IL7R	1.14
iCAFs	ALDH1A1	0.81	iCAFs	ANXA2	0.35	apCAFs	PCAT14	1.13
iCAFs	CCDC80	0.81	iCAFs	CRISPLD2	0.35	apCAFs	CLDN7	1.12
iCAFs	SFRP1	0.77	iCAFs	SH3BGRL	0.35	apCAFs	SLC45A3	1.1
iCAFs	PLAC9	0.75	iCAFs	INMT	0.34	apCAFs	ELF3	1.07
iCAFs	OGN	0.75	iCAFs	RORB	0.34	apCAFs	RACK1	1.07
iCAFs	TCEAL7	0.73	iCAFs	RARRES2	0.34	apCAFs	DDIT4	1.06
iCAFs	EFEMP1	0.73	iCAFs	ISL1	0.34	apCAFs	CD24	1.04

iCAFs	C7	0.73	iCAFs	EIF4A2	0.34	apCAFs	PRSS8	1.02
iCAFs	MGST1	0.7	iCAFs	LY6E	0.34	apCAFs	PPDPF	1
iCAFs	SPOCK3	0.69	iCAFs	DGUOK	0.33	apCAFs	SNHG8	0.99
iCAFs	FABP5	0.68	iCAFs	CPQ	0.33	apCAFs	TPD52	0.98
iCAFs	ID3	0.67	iCAFs	PEBP1	0.33	apCAFs	MT-ND3	0.97
iCAFs	CRYAB	0.64	iCAFs	NPC2	0.33	apCAFs	HSPA8	0.96
iCAFs	KLF4	0.63	iCAFs	QSOX1	0.33	apCAFs	FBP1	0.94
iCAFs	ID2	0.62	iCAFs	IFI35	0.32	apCAFs	TMC5	0.9
iCAFs	GPM6B	0.62	iCAFs	DIO3OS	0.32	apCAFs	VAMP8	0.83
iCAFs	TCF21	0.61	iCAFs	MYL6B	0.31	apCAFs	MT-CO2	0.83
iCAFs	S100A4	0.61	iCAFs	ALDH2	0.31	apCAFs	DNAJB1	0.81
iCAFs	PLTP	0.6	iCAFs	BLVRB	0.31	apCAFs	EEF2	0.79
iCAFs	LGALS3BP	0.6	iCAFs	AKR7A2	0.31	apCAFs	HSPA1A	0.78
iCAFs	ID1	0.59	iCAFs	SEC62	0.31	apCAFs	SYTL1	0.75
iCAFs	SOD3	0.58	iCAFs	SMPDL3A	0.3	apCAFs	MT-CO1	0.73
iCAFs	CST3	0.58	iCAFs	EMP3	0.3	apCAFs	JPT1	0.66
iCAFs	FXYD6	0.58	iCAFs	SCPEP1	0.3	apCAFs	HSPA1B	0.65
iCAFs	IGFBP6	0.57	iCAFs	ZFYVE21	0.3	apCAFs	OCIAD2	0.59
iCAFs	GADD45G	0.56	iCAFs	ST13	0.3	apCAFs	NACA	0.57
iCAFs	EPHX1	0.56						

Abbreviation: CAF, cancer-associated fibroblast; myCAFs, myofibroblast-like CAFs; iCAFs, immune and inflammatory CAFs; apCAFs, antigen-presenting CAFs; FC, fold change.
