# Supplementary material for: Single-cell and bulk RNA sequencing reveal cancer-associated fibroblast heterogeneity and a prognostic signature in prostate cancer
Source: Medicine (Baltimore). 2023 Aug 11;102(32):e34611. doi: 10.1097/MD.0000000000034611 (PMC10419654; doi:10.1097/MD.0000000000034611)

Supplementary Figure 6. Pseudotime analysis demonstrates the development trajectories of CAFs, which was delineated by the cell type (a,d), pseudotime (b), and patient origin (c).

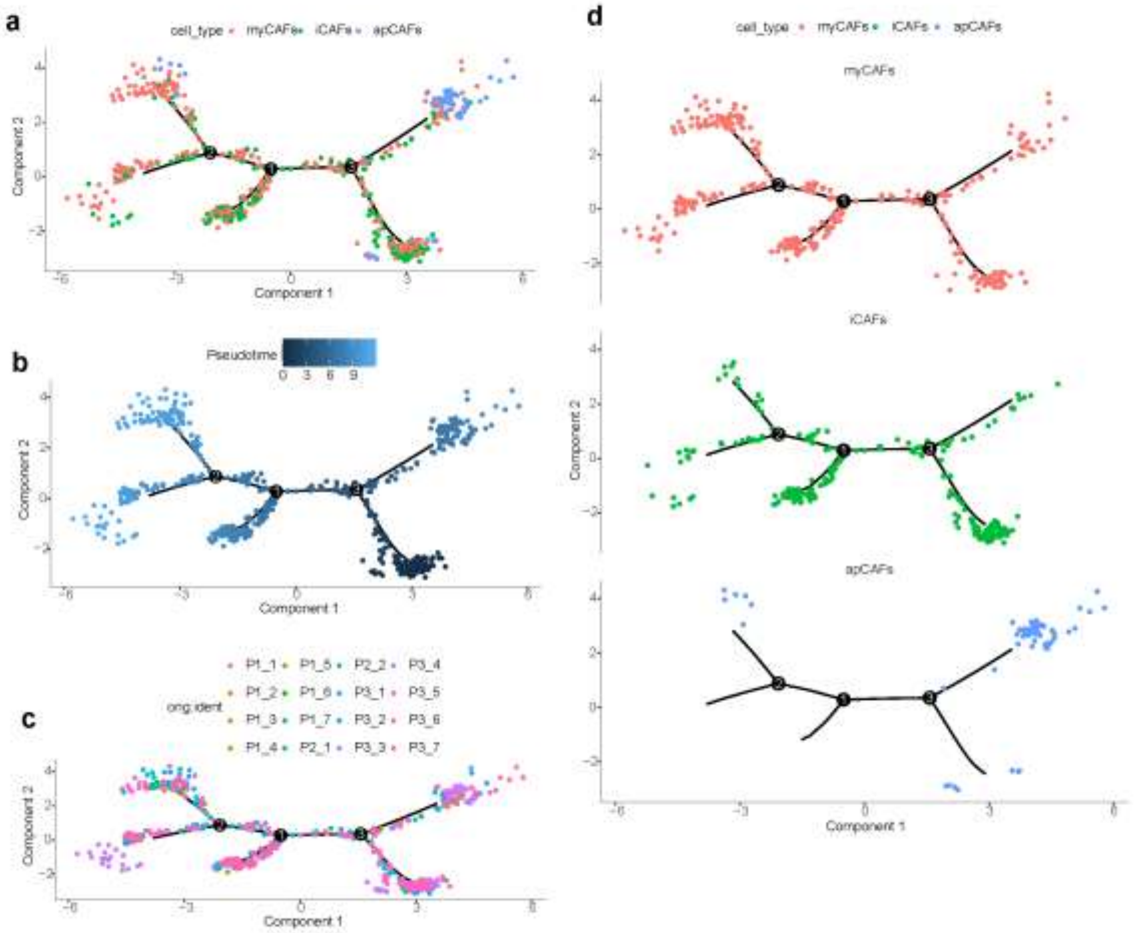

Supplement: Supplementary file 9 [file medi-102-e34611-s009.pdf]
